# Mapping the degenerating intervertebral disc: a systematic review of histological evidence

**DOI:** 10.3389/fmed.2026.1753988

**Published:** 2026-02-25

**Authors:** Francesca Veronesi, Francesca Salamanna, Giuseppe Tedesco, Alberto Ruffilli, Francesco Rosa, Cesare Faldini, Gianluca Giavaresi

**Affiliations:** 1 Surgical Sciences and Technologies, IRCCS Istituto Ortopedico Rizzoli, Bologna, Italy; 2 Department of Spine Surgery, IRCCS Istituto Ortopedico Rizzoli, Bologna, Italy; 3 1st Orthopaedic and Traumatologic Department, IRCCS Istituto Ortopedico Rizzoli, Bologna, Italy; 4 Department of Biomedical and Neuromotor Science-DIBINEM, University of Bologna, Bologna, Italy

**Keywords:** histological changes, immunohistochemical changes, intervertebral disc degeneration, molecular data, systematic review

## Abstract

**Introduction:**

Intervertebral disc degeneration (IDD) is a major cause of low back pain and disability. While MRI remains the standard diagnostic tool, it provides limited insight into the cellular and molecular changes underlying IDD. Histological analysis offers a complementary approach to characterizing the degenerative process in human intervertebral discs (IVDs). This systematic review aims to provide a comprehensive analysis of histological and immunohistochemical changes across the IVD, nucleus pulposus (NP), and cartilage endplate (CEP) in degenerated human discs.

**Methods:**

A literature search was conducted in PubMed, Scopus, and Web of Science for studies published between 2015 and 2025. A total of 45 human studies were included. Histological features, protein expression profiles, and grading systems were analyzed. Differentially expressed proteins were mapped into protein–protein interaction (PPI) networks using the STRING database.

**Results:**

Common histopathological features included ECM disorganization, proteoglycan depletion, fibrosis, neovascularization, and cell clustering. Molecular data revealed upregulation of catabolic enzymes, inflammatory cytokines, apoptotic mediators, and angiogenic factors. Conversely, regenerative and protective markers were significantly downregulated. PPI analysis revealed region-specific pathways: ECM remodeling and BMP/VEGF signaling in the IVD, inflammation and mechanotransduction in the NP, and ossification and prostaglandin signaling in the CEP.

**Conclusion:**

Histology reveals spatially distinct yet converging degenerative pathways across IVD regions. These findings identify potential biomarkers and therapeutic targets, supporting histological analysis as an essential complement to imaging for accurate IDD characterization.

## Introduction

Low back pain (LBP) is a leading cause of disability worldwide, affecting up to 80% of individuals at some point in their lives and imposing a significant socioeconomic burden (1). Among the primary causes of LBP, intervertebral disc degeneration (IDD) accounts for approximately 40% of cases (2, 3).

The intervertebral disc (IVD) is an avascular structure composed of three main components: (1) nucleus pulposus (NP), a central, gel-like region rich in proteoglycans (PGs), type II collagen (COLL II), elastin, and glycoproteins, responsible for load distribution and maintaining disc hydration; (2) annulus fibrosus (AF), a dense outer ring primarily made of type I collagen (COLL I), providing tensile strength and structural support; and (3) cartilage endplates (CEPs), a thin layers of hyaline cartilage that separate the disc from adjacent vertebral bodies and mediate nutrient and metabolite exchange (4–6).

The onset and progression of IDD are multifactorial, involving genetic predisposition, environmental factors, mechanical stress, aging, smoking, obesity, atherosclerosis, poor nutrition, and metabolic disorders (7, 8). A hallmark of IDD is the disruption of extracellular matrix (ECM) homeostasis, characterized by increased catabolic activity, driven by matrix metalloproteinases (MMPs), a disintegrin and metalloproteinase with thrombospondin motifs (ADAMTS) enzymes, and pro-inflammatory cytokines, such as interleukins (IL)1β, IL6, IL8, prostaglandin E2 (PGE2), and nitric oxide (NO), coupled with reduced anabolic signaling from factors like tumor necrosis factor *β* (TGFβ), bone morphogenetic proteins (BMPs), and insulin-like growth factor (IGF). This imbalance leads to PG depletion and water loss in the NP, followed by structural disruption of the AF, fissure formation, neovascularization, neoinnervation, and progressive cell apoptosis (9, 10). Recent studies have highlighted also the critical role of CEP degeneration in IDD progression. In fact, it was reported that increased calcification impairs the CEP’s ability to regulate nutrient diffusion and metabolic waste removal, contributing to local hypoxia, lactic acid accumulation, and potentially initiating the degenerative cascade (11, 12).

Diagnosis of IDD typically involves clinical evaluation supported by imaging, with magnetic resonance imaging (MRI) being the most sensitive non-invasive method for assessing disc morphology and hydration status (13). Among the MRI-based grading systems, the Pfirrmann classification focuses on NP and AF characteristics (14), while the Modic classification assesses signal changes in the CEP and adjacent vertebral bone marrow (15). However, MRI offers limited resolution in detecting early or subtle histopathological change, highlighting the mandatory complementary role of histological analysis, which provides high-resolution insights into cellular and ECM alterations underlying IDD (16, 17). Despite important contributions from animal models, the limited availability of human histological data continues to challenge the accurate characterization of disc degeneration and the development of targeted therapies.

The aim of this systematic review is to provide a comprehensively analysis of histological findings in human IVDs reported over the past decade. Specifically, we include studies investigating histological and immunohistochemical features associated with IVDs degeneration in human specimens, encompassing different tissues and stages of disease. When available, comparisons with non-degenerated (healthy) discs were also considered. To our knowledge, this is the first systematic review to focus exclusively on human specimens spanning various stages of degeneration.

## Methods

This systematic review was structured according to the PEO framework (Population, Exposure, Outcome). Eligible studies included those involving human IVDs of any degeneration severity (Population), with disc degeneration or degeneration grade as the exposure of interest, and histological and immunohistochemical findings as outcomes (Outcome). Studies were included regardless of the presence of a healthy control group (healthy IVDs), and both comparative and non-comparative study designs were considered.

On May 2025, a search was conducted in 3 databases (PubMed, Web of Science and Scopus) using the following string across all databases: (human intervertebral disc OR human intervertebral disc degeneration) AND (histology). The following filters were applied: a date ranges from 2015-05-01 to 2025-05-01, for PubMed and Web of Science, and 2015–2025 data range for Scopus.

**Figure 1:**
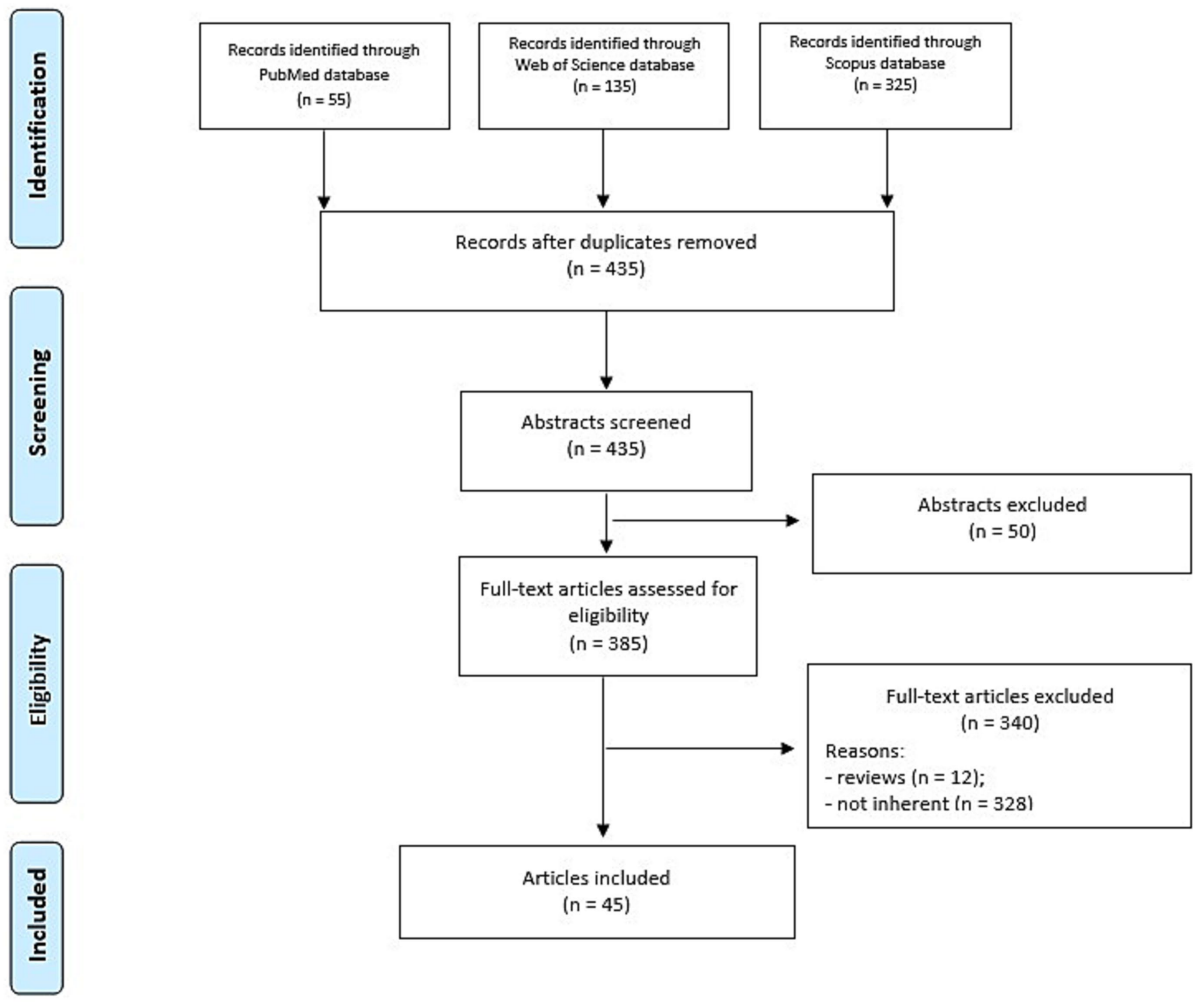
PRISMA flowchart of the study selection process.

The study selection process was illustrated using a flowchart, following the Preferred Reporting Items for Systematic Reviews and Meta-Analyses (PRISMA) guidelines, as shown in Figure 1.

Duplicate removal was performed using Rayyan, after which the remaining articles were screened based on their titles and abstracts according to the PEOinclusion criteria.

The following types of studies were excluded: preclinical studies, reviews, and studies focusing on no histological outcomes, involving IVD after pharmacological or physical treatment, and/or with not degenerated IVD. Subsequently, the full texts of articles were reviewed, in cases where abstracts did not provide sufficient information, and they were assessed using the inclusion and exclusion criteria. The article selection process was independently conducted by two authors (FV, FS), with disagreement on study eligibility resolved by a third author (GG).

Relevant data were independently extracted by two authors (FV, FS) and recorded in a standardized extraction form. The data collected included MRI-based and histological grading systems (Table 1), methodological overview of histological and immunohistochemical assessments (Table 2), experimental groups, diagnosis, grading scores, histology, results, and reference (Table 3).

**Table 1:** MRI-based and histological grading systems used in the included studies.

Study	Tissue	MRI grading system	Histological grading system
Hollenberg, 2021 (19)	IVD	Pfirrmann	NR
Lama, 2019 (20)	IVD	Pfirrmann	NR
Fan, 2022 (21)	IVD (NP, AF)	Pfirrmann	NR
Teixeira, 2021 (22)	NP	Pfirrmann	NR
Wei, 2025 (23)	IVD	Pfirrmann	NR
Rodrigues, 2019 (24)	IVD (NP, AF)	Pfirrmann	NR
Alvarez-Garcia, 2017 (25)	IVD	Thompson	NR
Otsuki, 2019 (26)	IVD	Thompson	NR
Cui, 2022 (27)	NP	NR	NR
Yang, 2018 (28)	IVD (AF, NP)	NR	NR
Yang, 2019 (29)	IVD	Pfirrmann	NR
Chen, 2019 (30)	IVD	Pfirrmann	NR
Ren, 2023 (31)	CEP	Thompson	NR
Yang, 2020 (32)	IVD	NR	NR
Li, 2018 (33)	IVD	Pfirrmann	NR
Lama, 2023 (34)	IVD	Pfirrmann	NR
Binch, 2015 (35)	IVD	NR	Sive et al.
Nakazawa, 2018 (36)	IVD	NR	Rutges et al.
Aras, 2016 (37)	IVD	Schneiderman	NR
Dube, 2025 (38)	IVD	NR	Sive et al.
Wang, 2021 (39)	NP	Pfirrmann	NR
Yu, 2022 (40)	IVD	Pfirrmann	NR
Zhu, 2025 (41)	IVD	Pfirrmann	NR
Liu, 2016 (42)	IVD	Pfirrmann	Sive et al.
Ionescu, 2024 (43)	IVD	Pfirrmann	NR
Guo, 2017 (44)	IVD	Pfirrmann	NR
Zhang, 2022 (45)	IVD	Pfirrmann	NR
Zhang, 2023 (46)	IVD	Pfirrmann	NR
Chen, 2022 (47)	IVD	NR	NR
Lian, 2017 (48)	IVD	Pfirrmann	NR
Liao, 2019 (49)	NP	Pfirrmann	NR
Zhan, 2024 (50)	IVD	Pfirrmann	NR
Tang, 2019 (51)	IVD	Pfirrmann	NR
Johnson, 2015 (52)	IVD	Sive et al.	NR
Li, 2025 (53)	IVD	Pfirrmann	NR
Wang, 2018 (54)	IVD	Thompson	NR
Yao, 2025 (55)	IVD	Pfirrmann	NR
Kang, 2017 (56)	IVD	Pfirrmann	NR
Zheng, 2025 (57)	IVD	Pfirrmann	NR
Jiang, 2019 (11)	CEP	Pfirrmann	NR
Ding, 2022 (58)	CEP	Thompson	NR
Huang, 2020 (59)	CEP	NR	NR
Huang, 2023 (60)	CEP	Pfirrmann	NR
Chen, 2024 (61)	IVD	Pfirrmann	NR
Bing, 2024 (62)	IVD	Pfirrmann	NR

NR, not reported.

**Table 2:** Methodological overview of histological and immunohistochemical assessments in human IVD degeneration studies.

Methodological aspect	Variants reported in included studies
Tissue source	Surgical specimens; cadaveric donors
Anatomical region	Whole IVD, nucleus pulposus, annulus fibrosus, cartilage endplate
Embedding medium	Paraffin; OCT; methacrylate (MMA)
Histological staining	H&E; Safranin O/Fast Green; Alcian Blue; Toluidine Blue; Picrosirius Red
Protein detection	Immunohistochemistry (brightfield); immunofluorescence
Detection system	DAB; alkaline phosphatase; fluorescence
Antibody type	Monoclonal or polyclonal (variously reported)
Quantification strategy	Qualitative localization; % positive cells; % positive area; semi-quantitative scoring (+/++/+++); mean fluorescence intensity

**Table 3:** Characteristics of the included studies on histological and immunohistochemical results on non-degenerated and degenerated IVD, NP and CEP.

Tissue	Groups	Diagnosis	Grading scores	Histology	Results	Ref.
IVD	1) 12 non-degenerated (6 M/ 6F, 14–66 yrs); 2) 36 degenerated (15 M/ 21F, 19–82 yrs)	1) Fractures tumor, scoliosis; 2) Herniation, spondylolisthesis, stenosis	Pfirrmann 1) I, II; 2) III, IV	Paraffin. IHC: BMP2, pSMAD1/5/8	Group 2: ↑ BMP2, pSMAD1/5/8 than group 1	Hollenberg, 2021 (19)
	1) 8 non-degenerated (8F, mean 14.5 yrs); 2) 21 degenerated (8 M/ 13F, mean 53 yrs)	1) Scoliosis; 2) Herniation	Pfirrmann 1) I; 2) III, IV	OCT. Staining: H&E, Toluidine blue. IHC: MMP1, denatrurated COLL I, II	Group 2: ↑ tears, GAG loss, cell clustering, MMP1, denaturated COLL I, II than group 1	Lama, 2019 (20)
	1) 6 non-degenerated; 2) 6 degenerated	1) Fractures; 2) Herniation	Pfirrmann 1) I, II; 2) IV, V	OCT. IF: RIP3, MLKL, pMLKL, MyD88	Group 2: ↑ RIP3, MLKL, pMLKL in NP and AF, MyD88 in NP than group 1	Fan, 2022 (21)
	1) 10 non-degenerated (2 M/ 8F, 17 ± 4 yrs); 2) 39 degenerated (15 M/ 24F, 63 ± 12 yrs)	1) Scoliosis; 2) compressive fracture, fall back surgery syndrome, osteochondrosis, herniation, spondylolisthesis, degenerative scoliosis, Chronic LBP, spinal canal stenosis,	Pfirrmann 1) I, II; 2) III-V	Paraffin. Staining: Safranin-O/Fast Green. IHC: TCC	Group 2: ↑ TCC in NP than group 1	Teixeira, 2021 (22)
	1) 5 non-degenerated (2 M/ 3F, 35.6 ± 9.5 yrs); 2) 5 degenerated (3 M/ 2F, 45 ± 6.9 yrs)	1) Fractures; 2) Herniation	Pfirrmann 1) I; 2) III-V	Paraffin. IHC: DEFB1	Group 2: ↑ DEFB1 than group 1	Wei, 2025 (23)
	1) 6 non-degenerated (4 M/ 2F, 33.8 ± 5.1 yrs); 2) 83 degenerated (51 M/ 32F, 36.4 ± 9.8 yrs)	1) Fractures; 2) LBP associated with radicular pain	Pfirrmann 1) I; 2) III, IV	Paraffin. IHC: IL6, CatB	Group 2: ↑ IL6, CatB in AF and NP than group 1	Rodrigues, 2019 (24)
	1) 4 non-degenerated (2 M/ 2F, 43 ± 3 yrs); 2) 4 degenerated (4 M, 62 ± 10 yrs)	1) From cadavers; 2) n.r.	Thompson 1) II; 2) IV	Paraffin. Staining: Safranin O-fast green. IHC: FOXO1, FOXO3	Group 1: ↑ FOXO1, FOXO3 than group 2. Group 2: fibrotic NP with some cell clusters, loss of NP/AF demarcation, small ruptures in the AF and general disorganization of AF lamellar structure	Alvarez-Garcia, 2017 (25)
	1) 4 non-degenerated (mean 43.3 yrs). 2) 4 degenerated (mean 62.0 yrs)	1) From cadavers; 2) n.r.	Thompson 1) II; 2) IV	Paraffin. Staining: Safranin O, Picrosirius red. IHC: SULF1, SULF2	Group 2: loss of GAG. Group 2: ↑ SULF1, SULF2 than group 1	Otsuki, 2019 (26)
	1) 3 non-degenerated (1 M/ 2F, 16.7 ± 3.1 yrs); 2) 3 degenerated (1 M/ 2F, 45.3 ± 7.3 yrs)	1) Scoliosis; 2) Herniation	n.r.	Paraffin IHC: MMPCC	Group 2: ↑ MMPCC in NP than group 1	Cui, 2022 (27)
	1) 8 non-degenerated (5 M/ 3F, 51.5 ± 8.8 yrs); 2) 52 degenerated (29 M/ 23F, 50.3 ± 7.8 yrs)	1) From cadavers; 2) Chronic LBP, intractable dizziness	n.r.	Paraffin. Staining: H&E. IHC: S100, SP	Group 2: ↑ Ruffini corpuscles, SP, S100 in the number and deeply ingrown into the inner AF and NP than group 1	Yang, 2018 (28)
	1) 485 non-degenerated (233 M/ 252F, median 54.2 yrs); 2) 454 degenerated (195 M/ 259F, median 51.6 yrs)	1) n.r.; 2) herniation, spinal stenosis, spondylolisthesis	Pfirrmann 1) I; 2) n.r.	Paraffin. IHC: VDR	Group 2: ↓ VDR than group 1	Yang, 2019 (29)
	88 IVD (43 M/45F, mean 45 yrs): 1) 20 non-degenerated; 2) 68 degenerated	1) Trauma or deformation; 2) Herniation	Pfirrmann 1) I, II; 2) III-V	Paraffin. IHC: PON1	Group 2: ↓ PON1 than group 1	Chen, 2019 (30)
	1) 7 non-degenerated (3 M/ 2F, 35 ± 5.5 yrs); 2) 22 degenerated (10 M/ 7F, 54 ± 15.8 yrs)	1) From cadavers; 2) Chronic LBP	Thompson 1) I, II; 2) III, IV	Paraffin. Staining: Safranin-O/Fast Green staining	Group 1: normal CEP transition pattern with abundant PG and compact collagen fiber. Group 2: ↓ PG in CEP, tissue calcification and necrosis	Ren, 2023 (31)
	1) 5 non-degenerated (19.6 ± 1.1 yrs); 2) 34 degenerated (20 M/ 14F, 51.2 ± 11.8 yrs)	1) Fractures; 2) Herniation or spondylolisthesis	n.r.	Paraffin. Staining: H&E, Safranin O. IHC: S1PR1, S1PR2, S1PR3	Group 2: ↓ S1PR1, S1PR2, S1PR3 than group 1	Yang, 2020 (32)
	1) 4 non-degenerated (2 M/ 2F, 8.8 ± 1.7 yrs); 2) 9 mildly degenerated (4 M/ 5F, 41.4 ± 13.7 yrs); 3) 4 severely degenerated (1 M/ 3F, 66 ± 2.6 yrs)	1) Scoliosis; 2), 3) Herniation	Pfirrmann 1) I, II; 2) III; 3) V	Paraffin. IHC: WNT5a, TNFα	Group 2: ↓ WNT5a; ↑ TNFα than groups 1, 2	Li, 2018 (33)
	1) 15 non-degenerated (6 M/ 9F, mean 53 yrs); 2) 19 mildly degenerated (6 M/ 13F, mean 53 yrs); 3) 21 severely degenerated (9 M/ 12F, mean 55 yrs)	1) From cadavers; 2) Scoliosis; 3) Herniation	Pfirrmann 1) II; 2) III; 3) IV	OCT. Staining: H&E, Toluidine blue. IHC: MMP1, Caspase3, Ki67, PCNA	Group 1: parallel and crimped collagen fibers, flattened fibroblast-like cells, absent cell clusters, ↑ PG, Ki67, PCNA; ↓ MMP1, Caspase3. Group 3: cell clusters, inflammatory cells, disrupted collagen lamellae. Group 3: ↓ PG, Ki67, PCNA; ↑ MMP3, Caspase3 than groups 1, 2	Lama, 2023 (34)
	1) 7 non-degenerated (30.8 ± 7 yrs); 2) 16 mildly degenerated (49.2 ± 17.7 yrs); 3) 21 severely degenerated (56.9 ± 19.5 yrs)	n.r.	Sive et al. 1) 9.7 ± 1.2 2) 5.7 ± 0.9 3) 3.5 ± 0.8	Paraffin. IHC: Sema3C, Sema3D, NRP2, PA1	Groups 2, 3: ↑ Sema3C, NRP2, PA1 than group 1. Groups 1, 3: ↑ Sema3D than group 2	Binch, 2015 (35)
	1) 3 mildly degenerated (2 M/ 1F, 8, 93, 53 yrs); 2) 4 moderately degenerated (3 M/ 1F, 44, 81, 93, 93 yrs); 3) 5 severely degenerated (5 M, 93, 93, 85, 85, 85 yrs)	1)-3) intact, non-herniated	Rutges et al. 1) 0, 2, 3; 2) 4, 6; 3) 8, 10	MMA. IHC and IF: CCR7, CD163, CD206	Groups 2, 3: ↑ CCR7, CD163, CD206 than group 1	Nakazawa, 2018 (36)
	56 IVD (28 M/28F, mean 47 yrs): 1) 2 mildly degenerated; 2) 35 moderately degenerated; 3) 19 severely degenerated	1)-3) Herniation	Schneiderman 1) I; 2) II; 3) III	Paraffin. IHC: MMP11	Group 3: ↑ MMP11 than groups 1, 2	Aras, 2016 (37)
	1) 18 mildly degenerated (8 M/ 10F, 43.4 ± 13.2 yrs); 2) 17 severely degraded (10 M/ 7F, 39.2 ± 5.9 yrs)	n.r.	Sive et al. 1) 4–7; 2) 10–12	Paraffin. Staining: H&E. IHC: AEBP1	Groups 1, 2: Cell clusters, loss of eosin staining, presence of fissures. Group 2: ↑ cell clusters, narrow fissures, AEBP1 than group 1	Dube, 2025 (38)
NP	1) 10 non-degenerated (4 M/ 6F, 15–25 yrs); 2) 18 degenerated (10 M/ 8F, 21–65 yrs)	1) Scoliosis; 2) Herniation	Pfirrmann 1) I-II; 2) III-V	Paraffin. IHC: PIEZO1	Group 2: ↑ PIEZO1 than group 1	Wang, 2021 (39)
	1) 49 non-degenerated (24 M/ 25F, 51.5 ± 9.2 yrs); 2) 65 degenerated (37 M/ 28F, 48.4 ± 9 yrs)	1) Fractures or scoliosis; 2) n.r.	Pfirrmann 1) 0; 2) II-V	Paraffin. IHC: ANG2	Group 2: ↑ ANG2 than group 1	Yu, 2022 (40)
	1) 17 non-degenerated (11 M/ 6F; 28.8 ± 7.9 yrs); 2) 17 degenerated (10 M/ 7F, 62.2 ± 12 yrs)	1) Scoliosis or fractures; 2) Herniation	Pfirrmann 1) I, II; 2) IV, V	Paraffin. Staining: H&E, Safranine-O/Fast green. IHC: POSTN, NLRP3, GSDMD-N, NOTCH1, IRF2	Group 2: ↑ fibrosis, aggregation of NP cells, POSTN, NLRP3, GSDMD-N, NOTCH1, IRF2; ↓ ECM than group 1	Zhu, 2025 (41)
	1) 3 non-degenerated (2 M/ 1F, 35 ± 12.5 yrs); 2) 7 degenerated (2 M/ 5F, 46.9 ± 11.4 yrs)	1) Fractures; 2) Herniation	Pfirrmann 1) I, II; 2) III-V	Paraffin. IHC: SDF1, CXCR4	Group 2: ↑ SDF1, CXCR4 than group 1	Liu, 2016 (42)
	1) 6 non-degenerated (1 M/ 5F, 14.2 ± 2.5 yrs); 2) 10 young degenerated (2 M/ 8F, 26.7 ± 5.9 yrs); 3) 11 middle-aged degenerated (1 M/ 10F, 48.3 ± 5.4 yrs); 4) 9 aged degenerated (1 M/ 8F, 73.2 ± 3.9 yrs)	1) Scoliosis; 2)-4) Chronic LBP	Sive et al. 1) 0; 2) 6.8 ± 3 3) 6 ± 2.8 4) 8.8 ± 3.6	Paraffin. Staining: H&E. IHC: CD24, TIE2	Group 4: ↑ CD24, TIE2 than group 1	Ionescu, 2024 (43)
	1) 4 non-degenerated (4 M, 36.3 ± 5.8 yrs); 2) 16 degenerated (7 M/ 9F, mean 60.8 ± 9 yrs)	1) Fractures; 2) Herniation	Pfirrmann 1) I, II; 2) III-V	Paraffin. Staining: H&E. IHC: SIRT1	Grade III: ↓ SIRT1 than grade II. Grade V: ↓ SIRT1 than grade IV. ↑ cell density; ↓ size clones from grade II to IV	Guo, 2017 (44)
	1) 12 non-degenerated (5 M/ 7F, 24.8 ± 11.1 yrs); 2) 12 degenerated (7 M/ 5F, 52.3 ± 18 yrs)	1) Trauma; 2) Herniation	Pfirrmann 1) I, II; 2) III-V	Paraffin. IHC: MTH1	Group 2: ↓ MTH1 than group 1	Zhang, 2022 (45)
	1) 2 non-degenerated (1 M/ 1F, 15 ± 1.4 yrs); 2) 8 degenerated (3 M/ 5F, 46.5 ± 19.8 yrs)	1) Scoliosis; 2) Herniation or spondylolisthesis	Pfirrmann 1) I; 2) II-V	Paraffin. Staining: H&E, Alcian blue. IHC: ENPP2, NOX4, FADS2	Group 2: ↑ NOX4; ↓ FADS2, ENPP2 than group 1	Zhang, 2023 (46)
	1) 3 non-degenerated; 2) 6 degenerated	1) Trauma; 2) Herniation	n.r.	Paraffin. IHC: COLL II, ACAN, ADAMTS4, TNF*α*	Group 2: ↓ COLL II, ACAN; ↑ ADAMTS4, TNFα than group 1	Chen, 2022 (47)
	1) 8 non-degenerated (8F, 14.3 ± 2.7 yrs); 2) 15 degenerated (4 M/ 11F, 56.9 ± 8.3 yrs)	1) Scoliosis; 2) n.r.	Pfirrmann 1) I, II; 2) IV, V	Paraffin. IHC: COLL II	Group 2: ↓ COLL II than group 1	Lian, 2017 (48)
	40 NP (16 M/24F, mean 32.5 yrs): 1) 10 non-degenerated; 2) 10 mildly degenerated; 3) 10 moderately degenerated; 4) 10 severely degenerated	1) Scoliosis; 2) Degenerative disc diseases	Pfirrmann 1) II; 2) III; 3) IV; 4) V	Paraffin. IF: ANGPTL8	Grade IV, V: ↑ ANGPTL8 than grade II, III	Liao, 2019 (49)
	1) 6 non-degenerated (3 M/ 3F, 28.3 ± 13.4 yrs); 2) 6 mildly degenerated (4 M/ 2F, 51.2 ± 10.2 yrs); 3) 6 moderately degenerated (4 M/ 2F, 56.2 ± 8.6 yrs); 4) 6 severely degenerated (3 M/ 3F, 63.5 ± 6.2 yrs)	n.r.	Pfirrmann 1) I; 2) III; 3) IV; 4) V	Paraffin. Staining: H&E. IHC: ADAMTS5, MMP3, MMP13, NLRP3, GSDMD, CASPASE1, IL1β, MINK1	↓ ECM content; ↑ ADAMTS5, MMP3, MMP13, NLRP3, CASPASE1, GSDMD, ILβ with degeneration progression. Group 2: ↑ MINK1. Groups 3, 4: ↓ MINK1	Zhan, 2024 (50)
	60 (mean 45.4 yrs): 1) 15 non-degenerated; 2) 15 mildly degenerated; 3) 15 moderately degenerated; 4) 15 severely degenerated	1)-4) LBP	Pfirrmann 1) I, II; 2) III; 3) IV; 4) V	OCT. IHC: NRF2	↓ NRF2 with degeneration progression	Tang, 2019 (51)
	1) 17 non-degenerated (13 M/ 4F, 38.1 ± 9.1 yrs); 2) 32 mildly degenerated (18 M/ 14F; 44 ± 14.1 yrs); 3) 31 severely degenerated (8 M/ 23F; 41 ± 12.8 yrs)	1) From cadavers; 2), 3) Prolapse	Sive *et al*. 1) 1–3.9; 2) 4–6.9; 3) 7–11	Paraffin Staining: H&E. IHC: AQPs 1, AQP 5	Groups 2, 3: ↓ AQP1, AQP5 than group 1	Johnson, 2015 (52)
	1) 7 mildly degenerated (3 M/ 4F, 43.4 ± 14.8 yrs); 2) 7 severely degenerated (5 M/ 2F, 48.6 ± 11.1 yrs)	1), 2) Herniation	Pfirrmann 1) II, III; 2) IV, V	Paraffin. Staining: Alcian blue, H&E. IF: PIEZO1	Group 2: ↑ PIEZO1 than group 2	Li, 2025 (53)
	20 (23 M/17F, 13–59 yrs): 1) 10 mildly degenerated; 2) 10 severely degenerated	1), 2) burst fracture, herniation, spinal stenosis, spondylolysis	Thompson 1) II, III; 2) IV, V	Paraffin. IHC: ANG2, COLL II, MMP13	Group 2: ↑ ANG2, MMP13; ↓ COLL II than group 1	Wang, 2018 (54)
	1) 5 mildly degenerated (4 M/ 1F, 33 ± 14.1 yrs); 2) 7 severely degenerated (5 M/ 2F, 43.9 ± 11.8 yrs)	1), 2) spinal deformity, lumbar spinal stenosis, spondylolisthesis, lumbar disc herniation, or spinal tumors	Pfirrmann 1) I, II; 2) III, IV	Paraffin. Saining: H&E, safranin O/fast green. IHC: LRP1	Group 2: ↓ LRP1 than group 1)	Yao, 2025 (55)
	20 (11 M/9F, mean 46.5 yrs): 1) 10 mildly degenerated; 2) 10 severely degenerated	1), 2) Chronic LBP	Pfirrmann 1) II, III; 2) IV, V	Paraffin. IHC: SOX9	Group 2: ↓ SOX9 than group 1	Kang, 2017 (56)
	1) 5 mildly degenerate (3 M/ 2F, 40.4 ± 13.4 yrs); 2) 5 moderately degenerate (2 M/ 3F, 40.6 ± 16.0 yrs); 3) 5 severely degenerate (3 M/ 2F, 62.6 ± 13.0 yrs)	1–3) herniation, lumbar spinal stenosis	Pfirrmann 1) II; 2) III; 3) IV	OCT. Staining: H&E, Safranin O/Fast Green. IHC: p16, EZH2, pSTING, IL1β, IL6	↑ structural looseness, PG loss, and tissue fibrosis in NP tissue with increase of degeneration. Group 2: ↑ p16, pSTING, IL1*β*, IL6; ↓ EZH2 than group 1 Group 3: ↑ p16, pSTING; ↓ EZH2 than groups 1, 2	Zheng, 2025 (57)
CEP	1) 4 non-degenerated fractured; 2) 14 degenerated	1) Fracture; 2) n.r.	Pfirrmann 1) I; 2) II, III, V	Paraffin. IHC: EZH2	Group 2: ↑ EZH2 than group 1	Jiang, 2019 (11)
	1) 4 non-degenerated (4F, 42.3 ± 5.4 yrs); 2) 6 severely degenerated (4 M/ 2F, 72.7 ± 2 yrs)	1) Scoliosis; 2) Spondylolisthesis, lumbar spinal stenosis, herniation	Thompson 1) I; 2) V	Paraffin. Staining: H&E, Alcian blu, Safranin O/Fast Green. IHC: YAP1, pYAP1, COLL II	Group 2: ↓ YAP1, COLL II; ↑ pYAP1 than group 1	Ding, 2022 (58)
	1) 15 non-degenerated (9 M/ 6F, mean 54.7 yrs); 2) 35 degenerated (20 M/ 15F, mean 60.9 yrs)	1) Burst fractures; 2) Chronic LBP	n.r.	Paraffin. Staining: H&E, Safranin O/Fast Green, Alcian Blue. IHC: MMP13, COLL II, Substance P, TNFα	Group 2: fibrotic and sclerotic ECM, fewer round chondrocytes, reduction in PG, CEP micro-damage. Group 2: ↑ MMP13, Substance P, TNF*α*; ↓ COLL II than group 1	Huang, 2020 (59)
	1) 6 non-degenerated (3 M/3F, 53.3 ± 12.7 yrs); 2) 7 mildly degenerated; 3) 8 moderately degenerated; 4) 6 severely degenerated	1) Burst fractures; 2) Chronic LPB	Pfirrmann 1) I, II; 2) III; 3) IV; 4) V	Paraffin. Staining: H&E, Alcian blue, Safranin O/fast green. IHC: COLL II. IF: NRF2	Group 4: ↓ COLL II, NRF2 than group 1	Huang, 2023 (60)
	71 degenererated (44 M/27F, mean 54.2 yrs): 1) 45 nondefect (27 M/ 18F, 2.5 yrs); 2) 26 defect (17 M/ 9 M, 57.2 yrs)	1), 2) Chronic LBP, herniation, spinalstenosis, degenerative spondylolisthesis	Pfirrmann 1) I, II (66.7%); IV, V (33.3%). 2) I, II (30.8%); IV, V (69.2%)	Paraffin. IHC: COX2, PGE2, EP4	Group 2: ↑ COX2, PGE2, EP4 than group 1	Chen, 2024 (61)
	23 (25 M/8F, 39.5 ± 6.9 yrs): 1) LVF (*n* = 7); 2) IDD (*n* = 16)		Pfirrmann 1) I, II; 2) III-VI	Paraffin. Staining: H&E, safranin O-fast green. IHC: P16, P21, MMP13, COLL I, OCN	Group 2: ↑ P16, P21 than group 1. Group 3: ↑ P16, P21, MMP13, COLLI, OCN than groups 1, 2	Bing, 2024 (62)

ACAN, Aggrecan; ADAMTS, A disintegrins, metalloproteinases with thrombospondin motifs; AEBP1, AE binding protein 1; AF, Annulus fibrosus; ANG2, Angiopoietin 2; ANGPTL8, Angiopoietin Like 8; AQP, Aquaporin; BMP2, Bone morphogenetic protein 2; CatB, CathepsinB; CEP, cartilage endplates; COLL, Collagen; COX2, Cyclooxygenase-2; CXCR4, C-X-C chemokine receptor type 4; DEFB1, Defensin beta 1; ECM, Extracellular matrix; ENPP2, Ectonucleotide Pyrophosphatase/Phosphodiesterase 2; EP4, Prostaglandin EP4 receptor; EZH2, Enhancer of zeste homolog 2; F, females; FADS2, Fatty Acid Desaturase 2; FOXO, forkhead box; GAG, glycosaminoglycans; GSDMD, Gasdermin D; H&E, Hematoxylin Eosin; IDD, Intervertebral disc degeneration; IF, Immunofluorescence; IHC, Immunohistochemistry; IL, Interleukin; IRF2, Interferon regulatory factor 2; IVD, Intervertebral disc; LBP, low back pain; LRP1, Low density lipoprotein receptor-related protein 1; LVF, lumbar fracture; M, males; MLKL, Mixed Lineage Kinase Domain Like; MMA, Methylmethacrylate; MMP, Metalloproteinase; MMPCC, MMP Cleaved C-terminus Aggrecan; MTH1, MutT homolog 1; n.r., not reported; NLRP3, NLR family pyrin domain containing 3; NOTCH1, Notch homolog 1, translocation-associated; NOX4, NADPH oxidase 4; NP, Nucleus pulposus; NRF2, Neuropilin 2; NRP, Nonribosomal peptides; OCN, Osteocalcin; OCT, optimal cutting temperature compound; PA1, Plexin A1; PCNA, Proliferating Cell Nuclear Antigen; PG, Proteoglycans; PGE2, Prostaglandin E2; PIEZO1, Piezo-type mechanosensitive ion channel component 1; PON1, Paraoxonase-1; POSTN, Periostin; RIP3, Receptor-Interacting Protein Kinase 3; S1PR, Sphingosine-1-Phosphate Receptor; SDF1, stromal cell-derived factor 1; Sema3, Semaphorin-3; SIRT1, Sirtuin; SMAD, small mother against decapentaplegic; SOX9, SRY-Box Transcription Factor 9; SP, Surfactant protein; STING, Stimulator of Interferon Genes; SULF, Sulfatase; TCC, Terminal complement complex; TIE2, Tyrosine-protein kinase receptor TEK; TNFα, Tumor necrosis factor α; VDR, Vitamin D receptor; WNT5a, Wnt Family Member 5a; YAP1, yes-associated protein 1; yrs, years.

The methodological quality and risk of bias of the included studies were assessed using the QUADAS-2 tool (Quality Assessment of Diagnostic Accuracy Studies-2; Table 4) (18). This tool evaluates four key domains: patient selection, index test, reference standard, and flow and timing. Each domain is assessed for risk of bias. The assessment was performed independently by two authors (FV and FS), and any discrepancies were resolved by consensus or through consultation with a third author (GG).

**Table 4:** QUADAS-2 risk of bias and applicability concerns.

Domain	Signaling questions	Risk of bias judgment
1. Patient Selection	1. Was a consecutive or random sample of patients enrolled? 2. Was a case–control design avoided? 3. Did the study avoid inappropriate exclusions?	Low / High / Unclear
2. Index Test	1. Were the index test results interpreted without knowledge of the reference standard results? 2. If a threshold was used, was it pre-specified?	Low / High / Unclear
3. Reference Standard	1. Is the reference standard likely to correctly classify the target condition? 2. Were the reference standard results interpreted without knowledge of the index test results?	Low / High / Unclear
4. Flow and Timing	1. Was there an appropriate interval between index test and reference standard? 2. Did all patients receive a reference standard? 3. Were all patients included in the analysis?	Low / High / Unclear

### Protein–protein interactions analysis

Protein–protein interactions (PPI) were analyzed using the Search Tool for the Retrieval of Interacting Genes/Proteins v11.5 (STRING; https://string-db.org, accessed on July 17, 2025), a database of physical and functional interactions. In the resulting network, nodes represent proteins and edges represent protein–protein associations. Protein clustering was conducted via K-means clustering (K = 3) within the STRING platform.

## Results

A total of 515 studies were identified (55 from PubMed, 135 from Web of Science, and 325 from Scopus). After removing 80 duplicates, 435 studies remained. Following abstract screening, 50 studies were excluded, and an additional 340 were excluded after full-text review due to being reviews (*n* = 12) or having methodological limitations (*in vitro/in vivo* models, lack of histological outcomes, or non-degenerated IVDs, pharmacological or physical treatment interventions, *n* = 328).

Ultimately, 45 studies were included in the systematic review (Table 3; Figure 1) (11, 19–62).

### Sample features

Most of the included studies analyzed IVD tissue (*n* = 20) (19–38), followed by NP (*N* = 19) (39–57), and CEP (*n* = 6) ones (11, 58–62).

The non-degenerated samples, employed as controls, were 800, that were compared with a total of 1,620 degenerated (ratio ~1:2).

Non-degenerated samples were obtained from cadavers (25, 26, 28, 31, 34, 52) or from patients undergoing surgery for trauma, scoliosis, or fractures. Degenerated samples were collected from patients with disc herniation, chronic LBP, spondylolisthesis, or spinal stenosis.

### Patient characteristics

The mean age of patients with non-degenerated IVDs was 33.8 ± 14.4 years, with an equal distribution between males and females (346 males, 378 females) while in patients with IVDs degeneration the mean age was 55.9 ± 17.7 years, with a comparable number of males and females participants (700 males, 719 females).

### MRI and histological grading systems

Three main MRI grading systems were used to assess degeneration of the IVDs, NPs, or CEPs:

- Pfirrmann grading (14) (Supplementary Table S1), applied in 29 studies (11, 19–24, 29, 30, 33, 34, 39–42, 44–51, 53–57, 60–62);
- Thompson grading (63) (Supplementary Table S2), used in 5 studies (25, 26, 31, 54, 58);
- Schneiderman classification (64) (Supplementary Table S3), used in 1 study (37).

Histological grading system were reported in 5 studies:

- The Sive et al. system (65), used in 4 studies (35, 38, 43, 52), ranges from 0 (normal) to 12 (severe degeneration; Supplementary Table S4).
- The Ritges et al. system (66), used in 1 study (36), scores six subcategories (AF, NP, EP) from 0 to 2, with a total score from 0 (healthy) to 12 (fully degenerated; Supplementary Table S5).

Five studies did not report any grading system (27, 28, 32, 47, 59).

### Overview of grading systems and histological protein assessments

To facilitate cross-study interpretation, the MRI-based and histological grading systems used in the included studies were systematically analyzed and are summarized in Table 1, which reports, for each study, the imaging and/or histological grading approaches adopted. Overall, MRI-based classifications were more frequently applied than histological grading systems and were predominantly used to assess whole IDD. Among these, the Pfirrmann grading system was the most commonly employed, followed by the Thompson and Schneiderman classifications, reflecting differences in study design, tissue availability, and resolution requirements. Histological grading systems were applied in a smaller subset of studies and enabled higher-resolution assessment of tissue architecture, cellular morphology, and matrix organization.

Importantly, structural grading systems describe morphological features of disc degeneration and are conceptually distinct from protein-level histological evaluations. Studies investigating molecular markers relied on heterogeneous experimental conditions, including differences in biopsy source and anatomical region, histological processing and embedding procedures, staining and detection methods, and quantification strategies. An overview of these methodological variables underlying histological and immunohistochemical protein assessments is provided in Table 2, which summarizes the major experimental categories reported across studies.

To further document methodological heterogeneity at the study level, detailed information on histological and immunohistochemical procedures is reported in Supplementary Table S6, which includes only studies performing protein-level assessments by immunohistochemistry or immunofluorescence. For each study, this table specifies tissue source, sample origin, embedding medium, staining approach, target proteins, and quantification strategy. Studies that did not perform protein-level histological analyses are therefore included in Table 1 but are not reported in Supplementary Table S6, reflecting the scope and methodological focus of each analysis.

### Histological processing

Histology was performed in all studies. Three different embedding media were used: paraffin (in 39 studies) (11, 19, 22–33, 35, 37–50, 52–56, 58–62), optimal cutting temperature compound (OCT; in 5 studies) (20, 21, 34, 51, 57), and methacrylate (MMA; in 1 study) (36).

As regards histological staining, the most commonly used was Hematoxylin and Eosin (H&E) (20, 28, 32, 34, 38, 41, 43, 44, 46, 50, 52, 53, 55, 57–60, 62), followed by Safranin O/Fast Green (22, 25, 26, 31, 32, 41, 55, 57–60, 62), Alcian Blue (46, 53, 58–60), Toluidine Blue (20, 34) and Picrosirius Red (26).

Immunohistochemistry (IHC) in brightfield (11, 19, 20, 22–30, 32–48, 50–52, 54–62) or fluorescence mode (21, 40, 49, 53, 60) was performed in almost all studies to detect and localize specific proteins or antigens within the tissue.

### Tissue analyses

#### IVD

Fourteen studies compared healthy and degenerated IVDs (19–32), while six examined tissues across varying grades of degeneration (33–38). Structural changes observed in degenerated IVDs included prevalently annular tears, PG loss, cell clustering, NP fibrosis, a wide-ranging of tissue disorganization, and presence of Ruffini corpuscles (20, 25, 26, 28, 31, 34, 38).

**Figure 2:**
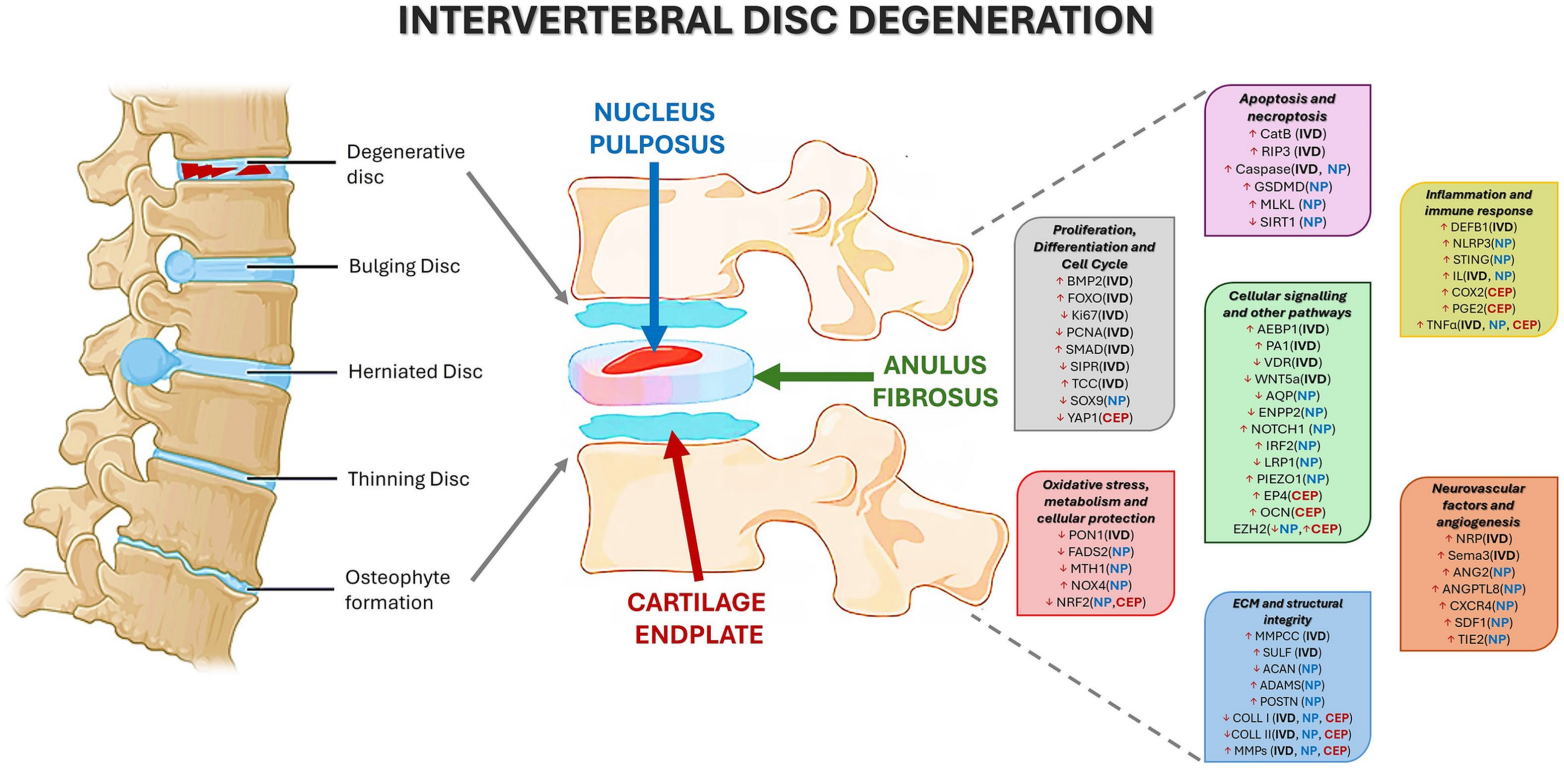
Protein up- and down-regulated in intervertebral disc degeneration, evaluated through immunohistochemistry.

Proteins upregulated in degenerated IVDs were classified according to their primary biological functions (Table 5; Figure 2), as follow reported:

1. *ECM and Structural Integrity*: ECM-degrading proteases (MMP1, MMP3, MMP11), aggrecan degradation marker (MMP-cleaved C-terminal aggrecan, MMPCC), i.e., a sulfation modulator of glycosaminoglycans (Sulfatase-SULF), and denatured types I and II collagen (COLL I, COLL II) (20, 26, 27, 34, 37);
2. *Neurovascular and Angiogenic Factors*: Semaphorin-3 (Sema3), a dual inducer of innervation and angiogenesis, and Neuropilin (NRP), a co-receptor for VEGF/Sema3 (35);
3. *Apoptosis and Necroptosis*: Apoptosis effector Caspase-3, necroptosis mediators RIP3, MLKL, and MyD88, and the lysosomal protease Cathepsin B (CatB), which contributes to apoptosis (21, 24, 34);
4. *Inflammation and Immune Response*: Pro-inflammatory cytokines (TNFα, IL6), and Defensin Beta 1 (DEFB1), a promoter of inflammation and senescence (23, 24, 33);
5. *Proliferation, Differentiation, and Cell Cycle*: Transcription factor FOXO, complement activator TCC, and signaling molecules in the BMP2/pSMAD1/5/8 pathway (19, 22, 25);
6. *Cellular Signaling and Other Pathways*: Neuronal and vascular signaling receptor PlexinA1 (PA1), and inflammation-associated transcription factor AEBP1 (35, 38);
7. *Macrophage Phenotype Markers*: CCR7, CD163, CD206 (36).

**Table 5:** Proteins, with their main activity/functions, up- and down-regulated in degenerated IVD, NP and CEP specimens.

Pathways involved	Protein	Main activity/function	IVD	NP	CEP
ECM and structural integrity	MMP	ECM degradation (protease)	↑	↑	↑
	MMPCC	C-terminal fragment of aggrecan (degradation indicator)	↑		
	SULF	Sulfate modulation in GAGs	↑		
	COLL (I, II)	Collagene strutturale del NP e AF	↓	↓	↓
	ADAMTS	ECM degradation (protease)		↑	
	POSTN	ECM remodeling and fibrosis		↑	
	ACAN	Major proteoglycan of the NP matrix		**↓**	
Neurovascular factors and angiogenesis	Sema3	Induction of nerve growth/angiogenesis	**↑**		
	NRP	Co-receptor for VEGF/Sema3, angiogenesis	**↑**		
	ANG2, ANGPTL8	Regulation of angiogenesis		**↑**	
	TIE2	Angiopoietin receptor tyrosine kinase		**↑**	
	CXCR4, SDF1	Chemotaxis, neoinnervation and vascularization		**↑**	
Apoptosis and necroptosis	RIP3, MLKL	Effectors of necroptosis	↑		
	CatB	Intracellular protein degradation, involved in apoptosis	↑		
	GSDMD	Effector of pyroptosis		↑	
	Caspase	Executor of apoptosis	**↑**	**↑**	
	SIRT1	Apoptosis reducer		↓	
Inflammation and immune response	IL	Pro-inflammatory cytokine	**↑**	**↑**	
	TNFα	Pro-inflammatory cytokine	**↑**	**↑**	**↑**
	DEFB1	Activates inflammation and senescence	**↑**		
	STING	Activation of the immune response		**↑**	
	NLRP3	Activates IL-1β and IL-18, innate inflammatory responses		**↑**	
	COX2	Enzyme for the synthesis of prostaglandins			**↑**
	PGE2	Vasodilation, inflammation, pain			**↑**
Proliferation, differentiation and cell cycle	FOXO	Transcription factor, stress/cell cycle	**↑**		
	TCC	Cell lysis by complement	**↑**		
	BMP2	Chondrogenic/osteogenic differentiation	**↑**		
	SMAD	TGF-β/BMP signal transducer	**↑**		
	Ki67	Proliferation marker	**↓**		
	PCNA	DNA replication marker	**↓**		
	S1PR	Regulation proliferation and migration	**↓**		
	SOX9	Transcription factor for chondrogenesis and ECM synthesis		**↓**	
	YAP1	Mechanotransduction, proliferation, regeneration			**↓**
Cellular signaling and other pathways	AEBP1	Inflammation-associated transcription factor	**↑**		
	WNT5a	Regulates development, cell polarity, non-canonical pathway	**↓**		
	PA1	Neuronal and vascular signaling	**↑**		
	VDR	Immune and bone regulation	**↓**		
	NOTCH1	Signaling and differentiation factor		**↑**	
	IRF2	Regulation of the antiviral/immune response		**↑**	
	PIEZO1	Mechanosensitive channel		**↑**	
	ENPP2	Phospholipid metabolism		**↓**	
	EZH2	Methyltransferase, epigenetic regulation		**↓**	**↑**
	AQP	Mechanosensitive channel Regulation of water transport (NP hydrostatics)		**↓**	
	LRP1	Protein clearance, modulation of cell signaling		**↓**	
	EP4	Mediator of the inflammatory effects of PGE2			**↑**
	OCN	Osteoblastic marker, bone regulation			**↑**
Oxidative stress, metabolism and cellular protection	PON1	HDL-Associated Antioxidant Enzyme	**↓**		
	NOX4	ROS generation		**↑**	
	NRF2	Master regulator of antioxidant response		**↓**	**↓**
	FADS2	Lipid metabolism		**↓**	
	MTH1	Protection from oxidative DNA damage		**↓**	

Differently, proteins found to be downregulated in degenerated IVDs included (Table 5; Figure 2):

1. *Proliferation, Differentiation, and Cell Cycle*: Ki67, DNA replication marker PCNA, and S1PR1, a regulator of proliferation and migration (32, 34);
2. *Cellular Signaling and Other Pathways*: Vitamin D receptor (VDR), and Wnt family member 5A (WNT5A), which regulates cell polarity and development (29, 33);
3. *Oxidative Stress, Metabolism, and Cellular Protection*: Antioxidant enzyme Paraoxonase-1 (PON1) (30).

Notably, the expression levels of key proliferation markers, including Ki67 and proliferating cell nuclear antigen (PCNA), were significantly reduced as the severity of IVD degeneration increased. This decline reflects impaired cellular turnover and a reduced regenerative capacity within the disc tissue, suggesting a diminished ability of resident cells to proliferate and maintain tissue homeostasis. In parallel, there was a pronounced upregulation of a wide array of catabolic, inflammatory, and apoptotic mediators, indicating a progressive shift toward a hostile and degradative tissue microenvironment.

Among the upregulated factors were MMP3, involved in the breakdown of ECM components, Caspase-3, a key effector enzyme in the execution phase of apoptosis, and Sema3, which plays a role in axonal guidance but is increasingly recognized for its involvement in inflammation and matrix remodeling. Additionally, elevated levels of plasminogen activator inhibitor-1 (PAI-1), a regulator of fibrinolysis and tissue remodeling, and C-C chemokine receptor type 7 (CCR7), associated with immune cell recruitment and chronic inflammation, were observed. The increased expression of macrophage-associated markers CD163 and CD206, indicative of alternatively activated (M2-like) macrophage infiltration, further supports the presence of an inflammatory immune response. Furthermore, MMP11 and adipocyte enhancer-binding protein 1 (AEBP1), both implicated in tissue remodeling, fibrosis, and inflammation, were significantly upregulated, collectively reinforcing the notion of a degenerative cascade characterized by enhanced matrix degradation, chronic inflammation, and apoptotic cell loss in advanced IVD degeneration (34–38).

#### Np

Ten studies compared healthy and degenerated NP tissues (39–48), while the remaining investigated graded degeneration (49–57). Degenerated NP tissues exhibited increased cell clustering, fibrosis, PG loss, and reduced cell density (41, 57).

Upregulated proteins were classified as follows (Table 5; Figure 2):

1. *ECM and Structural Integrity*: proteases (MMP3, MMP13), ECM-degrading enzyme ADAMTS4, and ECM remodeling/fibrosis mediator Periostin (POSTN) (41, 47, 50, 54);
2. *Neurovascular Factors and Angiogenesis*: Angiogenesis regulators ANGPTL8, ANG2, and TIE2, and chemoattractants/innervation inducers SDF1 and CXCR4 (40, 42, 43, 49);
3. *Apoptosis and Necroptosis*: Pyroptosis mediator Gasdermin D (GSDMD) and apoptosis effector Caspase-1 (41, 50);
4. *Inflammation and Immune Response*: Cytokines TNFα, IL6, IL1β, and innate immune activators STING and NLRP3 (41, 47, 50, 57);
5. *Cellular Signaling and Other Pathways*: Differentiation and signaling protein NOTCH1, immune regulator IRF2, and mechanosensitive channel PIEZO1 (39, 41, 53);
6. *Oxidative Stress, Metabolism, and Cellular Protection*: ROS producer NOX4 (46).

Downregulated proteins included (Table 5; Figure 2):

1. *ECM and Structural Integrity*: COLL II and Aggrecan (ACAN) (47, 48, 54);
2. *Apoptosis and Necroptosis*: Apoptosis suppressor Sirtuin 1 (SIRT1) (44);
3. *Proliferation, Differentiation, and Cell Cycle Regulation*: Chondrogenic transcription factor SOX9 (56);
4. *Cellular Signaling and Other Pathways*: Phospholipid metabolism enzyme ENPP2, methyltransferase EZH2, water channel proteins AQP1 and AQP5, and LRP1, a receptor involved in protein clearance (46, 52, 55, 57);
5. *Oxidative Stress, Metabolism, and Cellular Protection*: Antioxidant regulator NRF2, lipid metabolism enzyme FADS2, and oxidative DNA damage protector MTH1 (45, 46, 51).

Furthermore, the expression levels of several pro-degenerative and pro-inflammatory mediators, including angiopoietin-like 8 (ANGPTL8), a regulator of lipid metabolism and inflammation; ADAMTS5, a key aggrecanase implicated in cartilage matrix degradation; MMP3 and MMP13, both involved in the breakdown of ECM components; and components of the inflammasome pathway such as NLRP3, Caspase-1, and GSDMD, were significantly elevated in correlation with the increasing severity of intervertebral disc degeneration. Inflammatory cytokines, including IL-1β and IL-6, as well as mechanosensitive ion channel PIEZO1 and angiopoietin-2 (ANG2), further contributed to the inflammatory and catabolic microenvironment.

Additionally, elevated levels of STING (stimulator of interferon genes), a key mediator of innate immune responses and cellular senescence, were observed, highlighting the involvement of immune and stress-related pathways in disease progression.

In contrast, the expression of protective, homeostatic, and anabolic factors was markedly suppressed. These included NRF2, a master regulator of oxidative stress defense; aquaporins AQP1 and AQP5, which are essential for maintaining water homeostasis and cellular function, COLL II, LDL receptor-related protein 1 (LRP1), which plays a role in ECM turnover and inflammation resolution, SRY-box transcription factor 9 (SOX9), a key regulator of cartilage matrix production, and enhancer of zeste homolog 2 (EZH2), a histone methyltransferase involved in epigenetic regulation and stem cell maintenance. The downregulation of these factors underscores a critical imbalance between catabolic destruction and anabolic repair mechanisms, contributing to the progressive structural and functional deterioration of the IVD (49–57).

#### Cep

Three studies compared healthy and degenerated CEPs (11, 58, 59) while others evaluated degeneration by grade or presence of structural defects (60–62). Structurally, degenerated CEPs exhibited fibrotic and sclerotic ECM, reduced PG content, lower numbers of round chondrocytes, and micro-damage (59).

Upregulated proteins in degenerated CEPs included (Table 5; Figure 2):

1) *ECM and Structural Integrity*: ECM-degrading enzyme MMP13 (59);

2) *Inflammation and Immune Response*: Pro-inflammatory cytokine TNFα, Prostaglandin-synthesizing enzyme COX2, and inflammation mediator PGE2 (59, 61);

3) *Cellular Signaling and Other Pathways*: EZH2, and PGE2 receptor EP4, which mediates inflammatory responses (11, 61).

Downregulated proteins included (Table 5; Figure 2):

1. *ECM and Structural Integrity*: COLL II (58–60);
2. *Proliferation, Differentiation, and Cell Cycle Regulation*: Yes-associated protein 1 (YAP1), a key player in proliferation, regeneration, and mechanotransduction (58);
3. *Oxidative Stress, Metabolism, and Cellular Protection*: NRF2 (60).

In patients with advanced IDD (classified as High Level of Pathology, HLP), there was a marked upregulation of key markers associated with cellular senescence and tissue degeneration. These included the cyclin-dependent kinase inhibitors p16 and p21, both of which are well-known indicators of cellular aging and stress-induced growth arrest. Additionally, a significant increase was observed in the expression of COLL I, MMP13, and osteocalcin (OCN), a marker of osteoblastic differentiation. In contrast, levels of COLL II, and the transcription factor NRF2, a crucial regulator of antioxidant responses and cellular protection against oxidative stress, were significantly decreased as the severity of degeneration progressed (60, 62). These molecular alterations suggest a shift from a healthy phenotype toward a more fibrotic profile, accompanied by a decline in regenerative capacity and an increase in oxidative cellular damage.

### PPI analysis

PPI networks, generated through the STRING database, revealed distinct molecular signatures within the IVD, NP, and CEP compartments under degenerative conditions.

**Figure 3:**
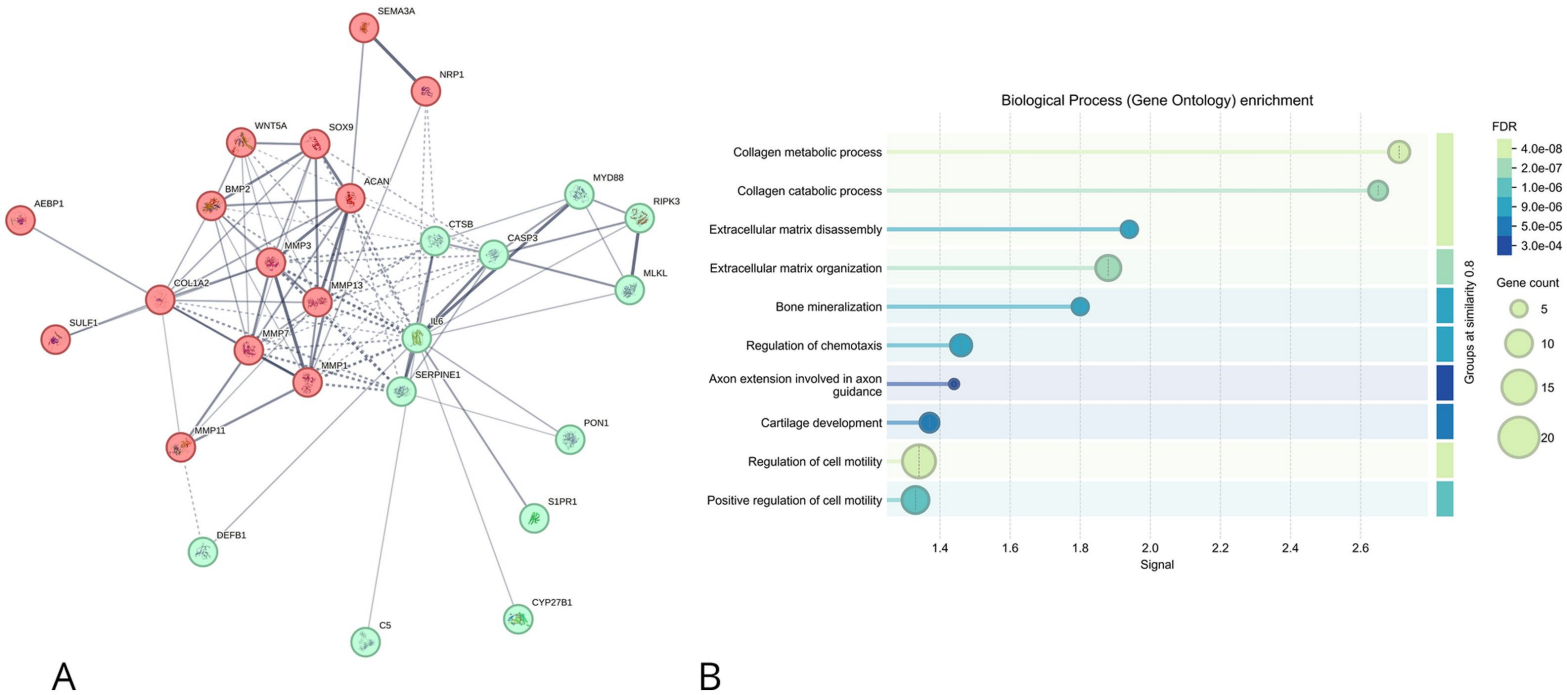
**(A)** STRING-generated protein–protein interaction (PPI) network of selected genes for IVD. Nodes represent proteins, and edges represent functional associations. Node color indicates functional grouping: red for extracellular matrix (ECM)–related proteins, green for inflammatory or cell death–related proteins, and others for regulatory elements. **(B)** Gene Ontology (GO) enrichment analysis (biological process category) of the genes shown in **(A)**. Dot plot displays top enriched GO terms ranked by signal strength, with dot size representing gene count per term and color indicating FDR-adjusted significance.

In the whole IVD (Figure 3A), ECM remodeling enzymes (e.g., MMPs), structural proteins (e.g., collagens), and regulatory transcription factors (e.g., SMADs, FOXO3) formed a tightly connected cluster. GO enrichment analysis (Figure 3B) highlighted biological processes such as collagen catabolism, cartilage development, and BMP/VEGF signaling, consistent with active tissue remodeling and angiogenesis.

**Figure 4:**
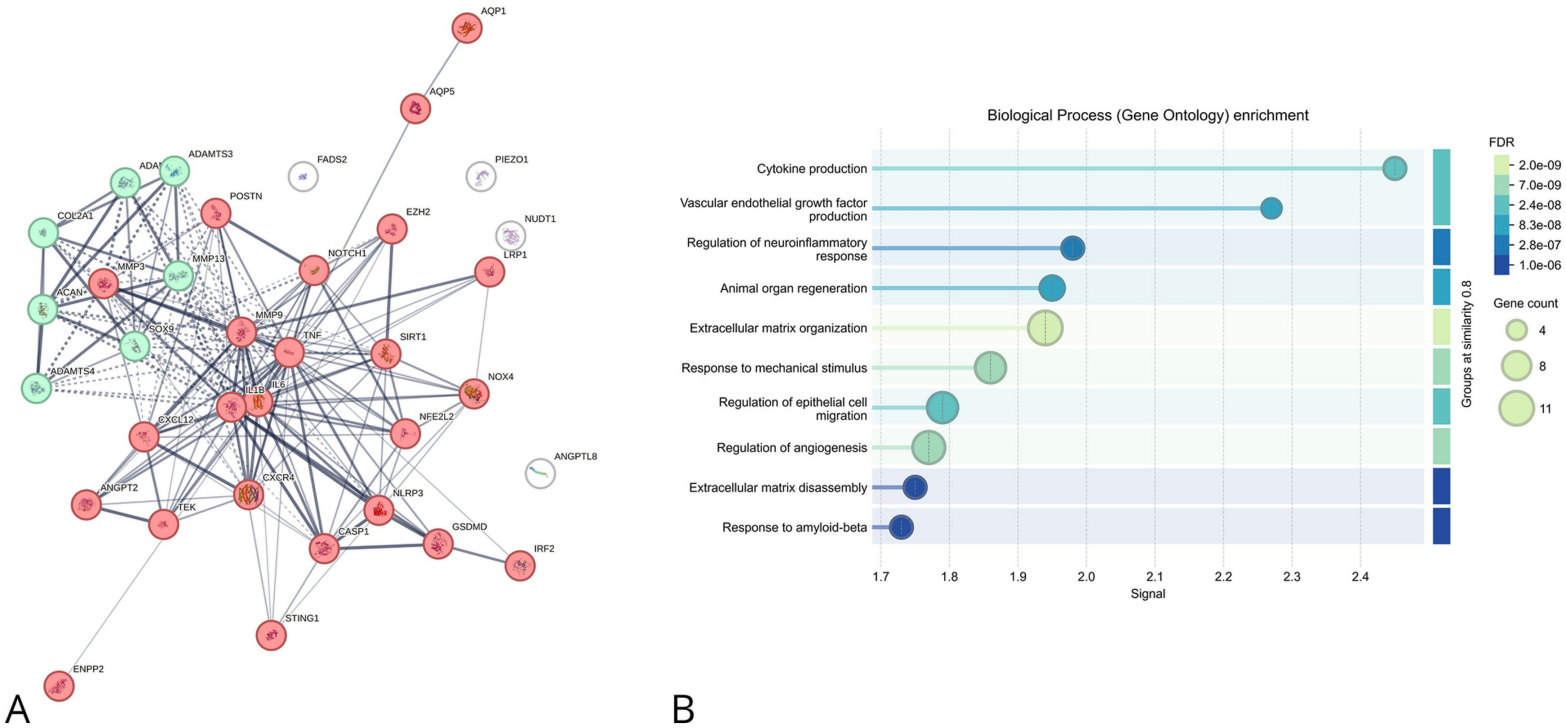
STRING-based protein–protein interaction (PPI) network and Gene Ontology (GO) enrichment analysis of differentially expressed proteins in nucleus pulposus (NP) from degenerated intervertebral disc. **(A)** STRING interaction network of inflammation- and ECM-associated proteins. Node colors and edge strengths represent interaction confidence and functional categorization. Key players such as CXCR4, IL6, TNF, and MMPs form a central highly connected cluster. **(B)** GO enrichment analysis reveals biological processes significantly represented among the genes, including cytokine production, extracellular matrix organization, and response to mechanical stimuli. Dot size and color scale reflect gene count and FDR significance, respectively.

In the NP (Figure 4A), the PPI network revealed strong interactions among inflammation-related proteins (e.g., TNF, IL6, CXCR4) and ECM regulators (e.g., MMP3, POSTN), indicating a predominant proinflammatory and catabolic environment. GO enrichment (Figure 4B) emphasized cytokine-mediated signaling, ECM disassembly, response to mechanical stress, and angiogenesis, supporting a scenario of sustained inflammatory damage and failed tissue regeneration.

**Figure 5:**
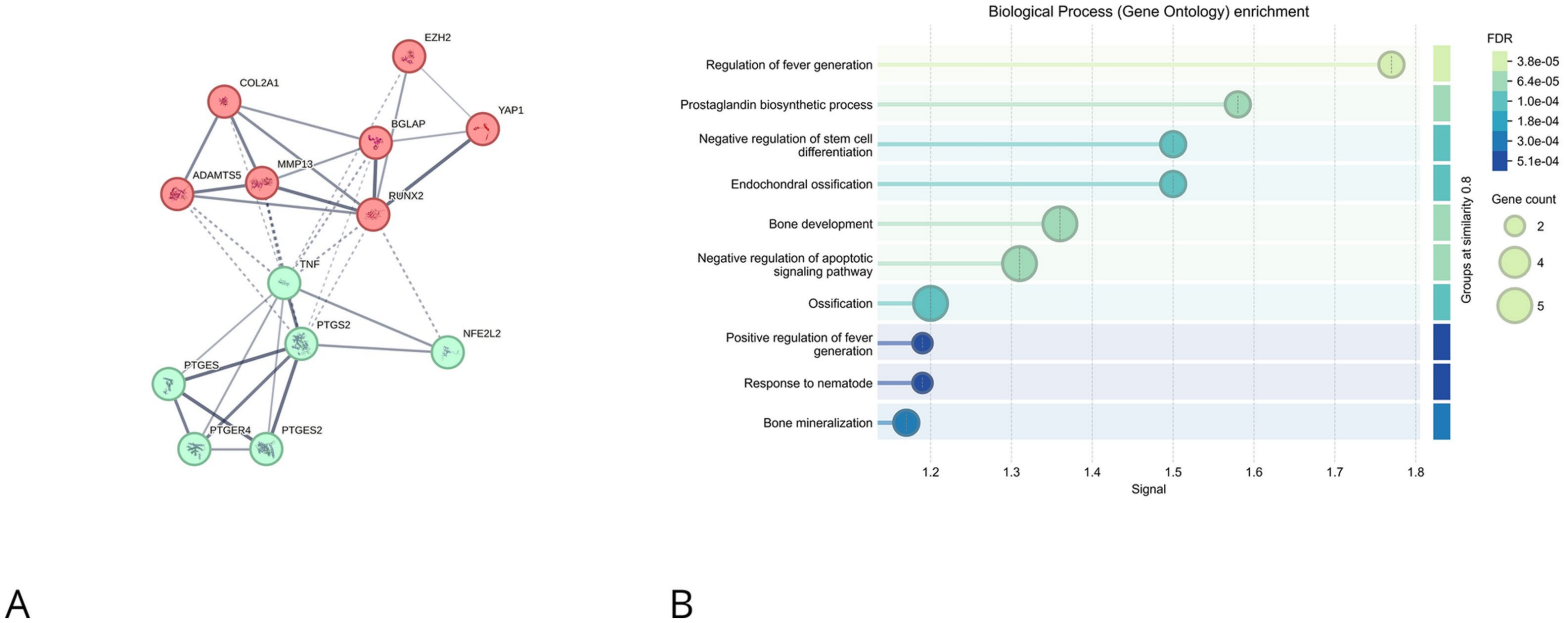
STRING-based protein–protein interaction (PPI) networks and Gene Ontology (GO) enrichment analyses of differentially expressed proteins in cartilage endplate (CEP). **(A)** Network analysis shows a subnetwork including PTGS2, PTGES, and osteogenic regulators (RUNX2, COL2A1, BGLAP). Interactions suggest a regulatory link between inflammation, prostaglandin metabolism, and ossification. **(B)** GO analysis highlights enriched biological processes such as prostaglandin biosynthetic process, bone development, and regulation of stem cell differentiation. Dot size and color scale reflect gene count and FDR significance, respectively.

The CEP (Figure 5A) exhibited a distinct subnetwork linking inflammatory mediators (e.g., PTGS2, EP4) with osteogenic factors (e.g., RUNX2, COL2A1, BGLAP). GO enrichment (Figure 5B) identified prostaglandin biosynthesis, ossification, and regulation of stem cell differentiation as major pathways, consistent with a shift toward an osteogenic and inflammatory phenotype. Collectively, the PPI analyses underscore spatially distinct yet converging degenerative mechanisms across IVD regions.

### Risk of bias assessment

**Figure 6:**
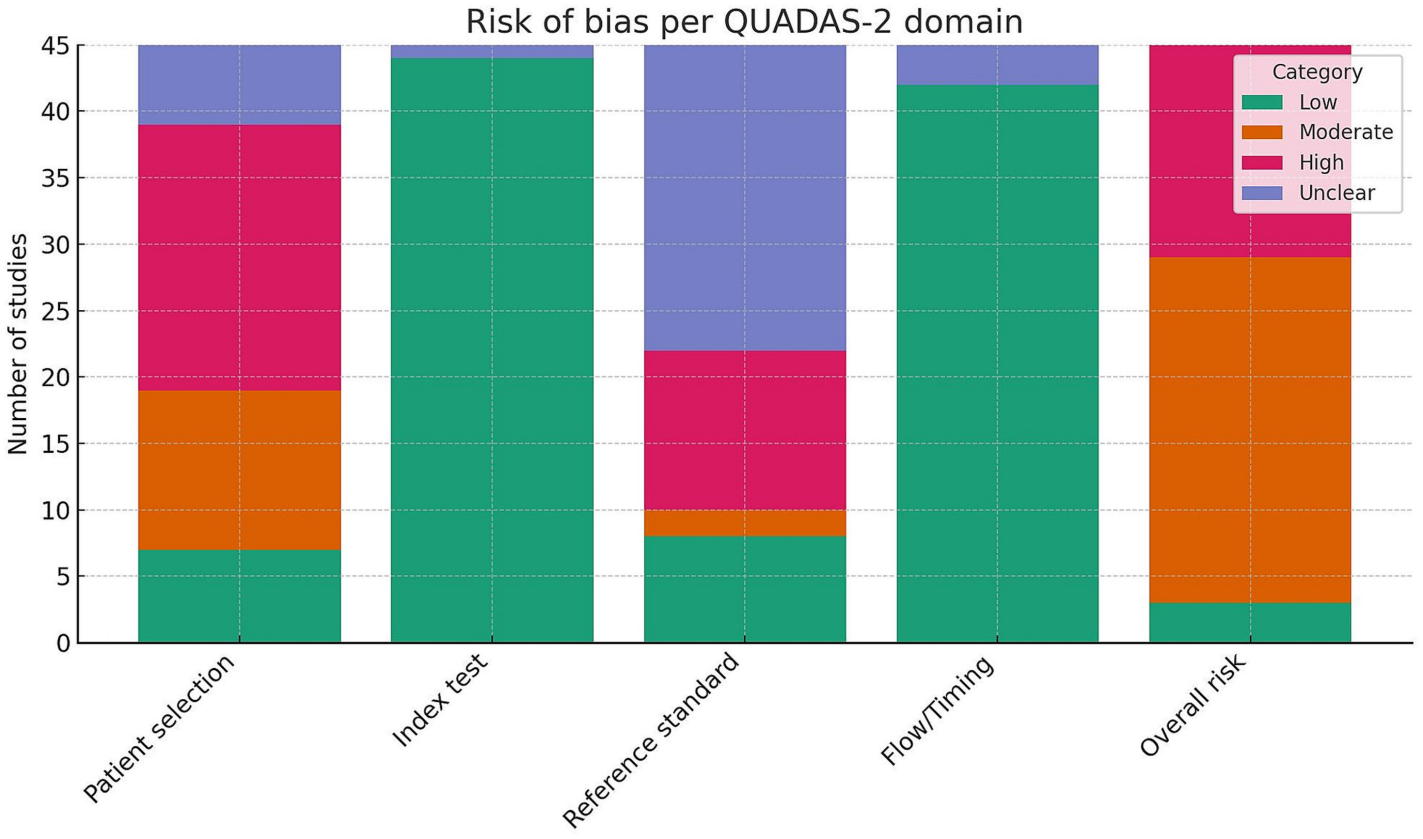
Summary of methodological risk of bias in included studies using the QUADAS-2 tool. The bar chart displays proportion of studies judged at low (green), moderate (orange), high (red), and unclear (violet) risk in each QUADAS-2 domain. While most studies showed low risk in the “index test” and “flow and timing” domains, concerns remained regarding patient selection and reference standard application.

Across the 45 eligible studies, patient-selection bias was the most frequent concern (Figure 6): only 7/45 (16%) were judged low risk, whereas 20/45 (44%) showed high and 6/45 (13%) unclear risk in this domain.

Conversely, the methodological description of the index tests was generally sound—44/45 studies (98%) met low-risk criteria, with just one study remaining unclear. The reference-standard domain proved critical: although 8 studies (18%) used a clearly validated standard (e.g., Thompson or Pfirrmann grading verified histologically), 12 (27%) were rated high risk and 23 (51%) unclear because of absent or insufficient detail. For flow and timing, nearly all investigations applied the same analytical pathway to every specimen; 42/45 (93%) were low risk and only three unclear. When the four domains were combined, overall risk of bias was low in 3 studies (7%), moderate in 26 studies (58%) and high in 16 studies (35%). These figures highlight that future research should prioritize transparent, prospectively defined inclusion criteria and adopt fully validated reference standards to strengthen methodological rigor.

## Discussion

This systematic review underscores the histological alterations observed in human IVDs across different stages of degeneration, providing a comprehensive reference framework that may support the identification of stage-specific biomarkers, guiding the development of targeted therapeutic strategies. While prior research has extensively utilized histological analyses on animal models to explore and analyze IDD degeneration (67, 68), this is the first systematic review that focus on human samples. Specifically, our review focused on three anatomical components, i.e., IVD, NP, and CEP, revealing specific trends in structural disruptions and molecular alterations.

Across the 45 included studies, a consistent pattern of histological changes was observed in degenerated IVDs. These included ECM disorganization, GAG and PG depletion, increased fibrosis, annular tears, cellular clustering, and signs of neovascularization and neoinnervation, particularly in the AF and CEP. These structural alterations reflect a progressive breakdown of the biomechanical and biochemical integrity of the IVD. Cell clustering, for instance, is considered a hallmark of degeneration and has been associated with attempts at cell proliferation in response to matrix degradation, although such attempts appear largely ineffective in halting the degenerative cascade (69). The demographic analysis revealed a clear age-related distinction between the non-degenerated and degenerated groups, with the former showing a significantly younger mean age compared to the latter (*p* < 0.001). This finding aligns with the well-established association between aging and IVD degeneration. Both groups exhibited a relatively balanced gender distribution. The origin of the samples also differed notably: non-degenerated discs were primarily obtained from cadavers or patients with conditions unrelated to degeneration, such as trauma, or fractures. Differently, degenerated samples were derived from patients with clinically diagnosed degenerative conditions, including disc herniation, chronic LBP, spondylolisthesis, or stenosis. These distinctions support the validity of sample classification and highlight the relevance of age and pathology in the progression of disc degeneration.

The molecular investigations performed on histological samples highlighted upregulation of catabolic enzymes (e.g., MMPs, ADAMTS4/5), inflammatory mediators (e.g., TNFα, IL-1β, IL-6), apoptotic and necroptotic regulators (e.g., Caspases and RIP3/MLKL), and neurovascular signaling factors (e.g., Sema3, ANG2, CXCR4). Conversely, anabolic, proliferation, and antioxidative mediators, such as SOX9, Ki67, PCNA, COL II, and NRF2, were markedly downregulated, especially with advanced degeneration. Severity-dependent associations suggested that molecular dysregulations intensify progressively as discs degenerate.

A central theme is the shift toward catabolism, driven by elevated protease activity (MMPs, ADAMTS) and inflammatory cytokines (ILs and TNFα). Impaired ECM integrity and loss of PG facilitate dehydration and compromise mechanical resilience. Downregulation of anabolic molecules such as SOX9, COL II, and ACAN further exacerbates structural decline. CEP calcification and diminished YAP1 likely restrict nutrient transport, aggravating hypoxic stress and undermining cellular viability. The interplay of inflammation and proteolysis underpins a self-reinforcing degenerative cycle that is evidently pronounced in advanced disease. Overexpression of catabolic and inflammatory factors is consistent with previous evidence indicating that inflammation is both a driver and a consequence of tissue breakdown (70, 71). Increased breakdown of ECM components is not countered by sufficient regenerative or anti-inflammatory signaling, leading to continued deterioration of disc structure and function (30).

Markers such as Caspase-3, −1, GSDMD, Cathepsin B, and RIP3/MLKL testify to simultaneous apoptosis, pyroptosis, and necroptosis within degenerated discs and elevated senescence-associated markers, including p16 and Defensin Beta 1, indicate accumulation of dysfunctional cells. These cell loss and senescence processes not only reduce functional cell pools but also contribute to pro-inflammatory secretomes and tissue dysfunction (72).

Upregulation of angiogenic mediators (ANG2, ANGPTL8, Sema3, NRP, CXCR4, SDF1, TIE2) and macrophage phenotypic markers (CCR7, CD163, CD206) reveals a marked gain in vascular and neural presence within degenerated disc layers. This aberrant innervation and neovascularization likely facilitate nociception and inflammation, linking structural breakdown with LBP symptoms (73).

Oxidative stress markers (e.g., NOX4) rose dramatically, in parallel with the diminishment of defense enzymes like PON1 and transcription factors such as NRF2. Depletion of mechanosensors (PIEZO1, AQP channels) and signaling regulators (WNT5a, VDR, EZH2, IRF2) suggests disruption of disc homeostasis, mechanosensitivity, and epigenetic equilibrium. These changes may impair cell physiology and response to mechanical load, accelerating degeneration (74).

Despite anatomical heterogeneity, IVD, NP, and CEP shared core degenerative patterns. However, CEP tissue showed a more pronounced inflammatory profile (e.g., COX2/PGE2) and mechanotransducive impairment (YAP1 reduction), likely linked to its unique nutrient-exchange role. CEP alterations may precipitate degeneration by compromising cellular nutrition, which subsequently amplifies NP/AF degradation. This layered evidence supports a model where CEP dysfunction initiates degeneration that cascades inward (75).

The PPI network analyses presented in this study provide a systems-level perspective on the molecular alterations occurring within distinct compartments of the degenerated IVD. Notably, our findings underscore the compartment-specific yet interrelated nature of the degenerative processes.

In the whole IVD, the emergence of a densely interconnected network, comprising MMPs, collagen isoforms, and signaling mediators such as SMADs and FOXO, reflects a coordinated activation of ECM remodeling pathways. The enrichment of biological processes related to collagen catabolism, cartilage development, and BMP/VEGF signaling aligns with the known hallmarks of IVD degeneration, including matrix degradation, neovascularization, and altered mechanotransduction.

Within the NP, the PPI network highlighted a dominant inflammatory signature, marked by strong interactions among cytokines (e.g., TNF, IL6) and their receptors (e.g., CXCR4), as well as ECM-modifying enzymes such as MMP3. GO enrichment further supported this proinflammatory milieu, pointing to sustained cytokine signaling, ECM disassembly, and angiogenic activity. These findings are consistent with prior studies indicating that chronic inflammation contributes to matrix breakdown and impairs regenerative potential in the NP.

Conversely, the CEP displayed a unique shift toward an osteogenic and proinflammatory phenotype, with prominent interactions involving prostaglandin-related enzymes (e.g., PTGS2, EP4) and osteogenic transcription factors (e.g., OCN). GO terms associated with ossification, prostaglandin biosynthesis, and regulation of stem cell differentiation suggest that CEP degeneration may involve endochondral-like ossification processes, potentially impairing nutrient diffusion and exacerbating disc pathology.

Together, these data reinforce the concept that IVD degeneration is not a uniform process, but rather involves spatially defined molecular responses that converge toward a dysfunctional and inflammatory tissue state. Understanding these regional differences is critical for the development of targeted therapies aimed at restoring disc homeostasis and halting disease progression.

One of the challenges highlighted by this review is the methodological heterogeneity across studies. A variety of histological grading systems were employed, including the Sive et al. (35, 38, 43, 52) and Ritges et al. (36) systems, and, similarly imaging assessments relied predominantly on the Pfirrmann classification (11, 19–24, 29, 30, 33, 34, 39–42, 44–51, 53–57, 60–62) although the Thompson et al. (25, 26, 31, 54, 58) and Schneiderman et al. (37) systems were also used. This lack of standardization complicates direct comparison of findings and underscores the need for more uniform criteria in future research. Most studies used hematoxylin and eosin staining as a primary diagnostic staining (20, 28, 32, 34, 38, 41, 43, 44, 46, 50, 52, 53, 55, 57–60, 62), often complemented by Safranin O/Fast Green (22, 25, 26, 31, 32, 41, 55, 57–60, 62) or Alcian Blue (46, 53, 58–60) for matrix evaluation, and IHC for protein quantification in all studies.

Beyond the biological findings, this systematic review provides a detailed methodological mapping of human intervertebral disc histology studies, which represents a key element of its originality. In contrast to previous reviews that primarily focused on molecular pathways or experimental models, the present work systematically integrates information on tissue source, anatomical localization, histological processing, immunohistochemical methodologies, and clinical characteristics of the studied populations (74–76).

Considerable heterogeneity emerged in antibody selection, staining protocols, and quantification approaches across studies. While most investigations relied on qualitative or semi-quantitative immunohistochemical assessments, only a limited number employed standardized scoring systems or digital image analysis for signal quantification. Differences were also observed in data representation, with results variably reported as percentage of positive cells, staining intensity, or descriptive localization, limiting direct cross-study comparisons. Anatomical localization represented an additional source of variability. Most studies focused on lumbar intervertebral discs, whereas cervical specimens were less frequently analyzed, often in distinct clinical contexts. Moreover, biopsy origin within the disc (whole IVD, nucleus pulposus, annulus fibrosus, or cartilage endplate) differed substantially among studies, influencing both histological appearance and molecular readouts.

Clinical heterogeneity further contributed to variability, as degenerated samples were obtained from patients with diverse diagnoses, including disc herniation, chronic low back pain, spinal stenosis, and spondylolisthesis, while non-degenerated controls were frequently derived from cadaveric donors or patients undergoing surgery for trauma or deformity. Importantly, the timing of tissue collection relative to imaging assessment or symptom duration was inconsistently reported, representing a critical limitation for the interpretation of degeneration-stage–specific findings.

By systematically capturing and synthesizing these methodological dimensions, the present review provides a structured framework that enables more informed interpretation of histological data and highlights key parameters that should be standardized in future human disc studies to improve reproducibility and translational relevance.

Despite variability in methodological approaches, the consistency in reported histological features across studies and tissue types strengthens the reliability of the findings. Additionally, the risk of bias assessment revealed overall moderate risk of bias across several domains. Most studies presented a low risk in patient selection, especially those using surgical samples with well-defined diagnostic criteria. Nevertheless, a number of studies lacked transparency regarding blinding procedures during histological and IHC analysis, introducing a potential risk of bias in the “index test” domain. Moreover, the absence of standardized histological grading systems in some studies, along with variability in sample preservation and processing protocols, may have affected internal validity and reproducibility. Additionally, flow and timing concerns emerged when the interval between MRI-based grading and histological analysis was not clearly reported. Despite these limitations, the consistency of the reported degenerative markers across independent cohorts reinforces the validity of the main conclusions.

The findings from this review have several clinical implications. The identification of key molecular actors offers potential biomarkers and therapeutic targets. Elevated MMPs, pro-inflammatory cytokines, and angiogenic factors could serve as stage-specific degeneration indicators, potentially refine diagnostic accuracy beyond MRI. Therapeutically, strategies aimed at restoring anabolic pathways (e.g., SOX9, COL II), enhancing antioxidative capacity (e.g., NRF2 modulation), and inhibiting catabolic/inflammatory signaling (e.g., targeting ADAMTS, MMPs, IL-1β/TNFα or Sema3) warrant exploration. Preserving CEP integrity and transport function (e.g., reducing calcification, upregulating YAP1) may offer early intervention avenues.

Several are the strength of the present systematic review. Analyzing the histology of the human IVD provides direct insight into the cellular and structural changes associated with IVD degeneration in its physiological biological context and offers distinct advantages over relying exclusively on animal models, particularly in translating findings to clinical practice. Human histological studies capture the authentic tissue microenvironment, including native inflammatory responses, ECM remodeling, and cellular phenotypes within the context of age-related degeneration. In contrast, animal models, though useful for mechanistic and interventional experiments, present significant species differences in spinal anatomy, disc composition, mechanical loading, and immune response, which can limit the extrapolation of results to humans (76). Additionally, quadrupedal biomechanics in most animal models differ markedly from the bipedal forces experienced by the human lumbar spine, making it challenging to replicate physiological loading conditions accurately (77). Human tissue analysis also circumvents ethical concerns, lifespan limitations, and standardization issues inherent in animal research. Ultimately, direct histological evaluation of human IVDs not only complements preclinical data but also ensures greater clinical relevance in understanding degenerative disc disease.

However, some limitations must be acknowledged. First, most of the included studies were cross-sectional in nature, limiting causal inferences about the sequence of degenerative changes. Second, variations in tissue procurement, processing, and staining techniques could introduce bias or artifacts. Moreover, healthy control samples were often derived from cadaveric donors or patients undergoing surgery for unrelated conditions (e.g., trauma), which may not perfectly represent truly healthy discs. Additionally, many studies did not report complete clinical information about the donors, such as comorbidities, smoking status, or physical activity levels, factors known to influence disc health. The heterogeneity in patient age across groups may also confound interpretation, as age-related changes may overlap with pathological degeneration.

## Conclusion

This systematic review consolidates current histological and molecular evidence from human IVD samples, highlighting the progressive and compartment-specific nature of degenerative changes. By integrating structural, cellular, and protein-level alterations, our findings reveal a consistent pattern of matrix disorganization, inflammation, vascularization, and cell death across degenerated discs, with region-specific molecular signatures in the NP, and CEP. The observed upregulation of catabolic enzymes, inflammatory cytokines, and pro-angiogenic factors, alongside the downregulation of anabolic, antioxidative, and mechanosensitive pathways, underscores a multifactorial degenerative cascade that worsens with disease severity.

Despite methodological heterogeneity, the convergence of histological markers across independent studies strengthens the validity of these findings and supports their translational relevance. Importantly, this review emphasizes the unique value of human tissue analysis over animal models, offering clinically meaningful insights into disc biology in the context of aging and pathology.

The identification of key degenerative mediators offers promising opportunities for biomarker discovery and targeted therapeutic development. Interventions aimed at modulating inflammation, enhancing matrix synthesis, preserving CEP function, and counteracting oxidative stress may represent viable strategies for delaying or reversing disc degeneration. Future research should prioritize longitudinal human studies, standardize histological grading protocols, incorporate comprehensive clinical metadata, and integrate histological findings with functional and mechanistic experiments to validate the biological relevance of the identified pathways and support their translational application in degenerative disc disease.

## Data Availability

The original contributions presented in the study are included in the article/Supplementary material, further inquiries can be directed to the corresponding author.
