# Supplementary material for: Mapping the degenerating intervertebral disc: a systematic review of histological evidence
Source: Front Med (Lausanne). 2026 Feb 25;13:1753988. doi: 10.3389/fmed.2026.1753988 (PMC12977079; doi:10.3389/fmed.2026.1753988)
Supplement: Supplementary file 1 [file Supplementary_file_1.pdf]

Supplemental data

Table S1. Pfirrmann grading system.

Grade	Structure	Distinction of Nucleus and Anulus	Signal Intensity	Height of Intervertebral Disc
I	Homogeneous, bright white	Clear	Hyperintense, isointense to cerebrospinal fluid	Normal
II	Inhomogeneous with or without horizontal bands	Clear	Hyperintense, isointense to cerebrospinal fluid	Normal
III	Inhomogeneous, gray	Unclear	Intermediate	Normal to slightly decreased
IV	Inhomogeneous, gray to black	Lost	Intermediate to hypointense	Normal to moderately decreased
V	Inhomogeneous, black	Lost	Hypointense	Collapsed disc space

Table S2. Thompson grading system.

Grade	Nucleus	Annulus	Endplate	Vertebral body
I	Bulging gel	Discrete fibrous lamellas	Hyaline, uniformly thick	Margins rounded
II	White fibrous tissue peripherally	Mucinous material between lamellas	Thickness irregular	Margins pointed
III	Consolidated fibrous tissue	Extensive mucinous infiltration; loss of annular-nuclear demarcation	Focal defects in cartilage	Early chondrophytes or osteophytes at margins
IV	Horizontal clefts parallel to endplate	Focal disruptions	Fibro cartilage extending from subchondral bone, irregularity and focal sclerosis in subchondral bone	Osteophytes less than 2 mm
V	Clefts extend through nucleus and annulus	–	Diffuse sclerosis	Osteophytes greater than 2 mm

Table S3. Schneiderman classification system.

Loss of demarcation between NP and AF	Loss of proteoglycan from NP	Presence and extent of fissure	Cell cluster formation
0 = Clear demarcation	0 = No loss of haematoxophilia	0 = No fissures	0 = No cell clusters
1 = Limited loss of demarcation	1 = Limited loss of haematoxophilia	1 = Fissures present within NP	1 = Less than 25% of cells formed into clusters
2 = Substantial loss of demarcation	2 = Substantial loss of haematoxophilia	2 = Fissures extending to junction of NP and AF	2 = 25–75% of cells formed into clusters
3 = Complete loss of demarcation	3 = Complete loss of haematoxophilia	3 = Fissures extending to within AF	3 = Over 75% of cells formed into clusters

Table S4. Sive *et al*. system.

Endplate	Morphology AF	Boundary AF and NP	Cellularity NP	Matrix NP	NP matrix staining
0 = Homogeneous structure; regular thickness	0 = Well-organized, half ring-shaped structure, collagen lamellae	0 = Clear boundary between AF and NP tissue	0 = Normal cellularity; no cell clusters	0 = Well-organized structure of nucleus matrix	0 = Intense staining; red stain dominates
1 = Slight irregularity with limited number of microfractures and locally decreased thickness	1 = Partly ruptured AF; loss of half ring-shaped structure	1 = Boundary less clear; loss of annular-nuclear demarcation	1 = Mixed cellularity; normal pattern with some cell clusters	1 = Partly disorganised structure of nucleus matrix	1 = Reduced staining; mixture of red and slight green staining
2 = Severe irregularity with multiple microfractures of the EP and generalized decreased thickness	2 = Completely ruptured AF; no intact half ring-shaped collagen lamellae	2 = No distinguishable boundary between AF and NP tissue	2 = Mainly clustered cellularity, chondroid nests present	2 = Complete disorganisation and loss of nucleus matrix	2 = Faint staining; increased green staining

Table S5. Ritges *et al*. system.

**Grade 0**	Normal (Hyperintense nucleus pulposus signal)
**Grade I**	Slight reduction in nucleus pulposus signal
**Grade II**	Global reduction in nucleus pulposus signal
**Grade III**	Hypointense nucleus pulposus signal and disc space narrowing

Table S6. Study-level methodological details of histological and immunohistochemical protein assessments. The Table includes only studies performing protein-level histological assessments by immunohistochemistry and/or immunofluorescence; studies limited to conventional histology without molecular/protein targets (e.g., Ren, 2023) are not included.

Study (Author, year)	Tissue analyzed	Sample source	Embedding	Staining / detection	Target proteins
Hollenberg, 2021	IVD	Surgical (fractures/tumor/scoliosis vs degeneration)	Paraffin	IHC (brightfield)	BMP2, pSMAD1/5/8
Lama, 2019	IVD	Surgical (scoliosis vs herniation)	OCT	Staining: H&E, Toluidine blue; IHC (brightfield)	MMP1, denatrurated COLL I, II
Fan, 2022	IVD (NP, AF)	Surgical (fractures vs herniation)	OCT	IF	RIP3, MLKL, pMLKL, MyD88
Teixeira, 2021	NP	Surgical (scoliosis vs mixed degenerative diagnoses)	Paraffin	Staining: Safranin-O/Fast Green; IHC (brightfield)	TCC
Wei, 2025	IVD	Surgical (fractures vs herniation)	Paraffin	IHC (brightfield)	DEFB1
Rodrigues, 2019	IVD (AF, NP)	Surgical (fractures vs LBP with radicular pain)	Paraffin	IHC (brightfield)	IL6, CatB
Alvarez-Garcia, 2017	IVD	Cadaveric controls; degenerated n.r.	Paraffin	Staining: Safranin O-fast green; IHC (brightfield)	FOXO1, FOXO3
Otsuki, 2019	IVD	Cadaveric controls; degenerated n.r.	Paraffin	Staining: Safranin O, Picrosirius red; IHC (brightfield)	SULF1, SULF2
Cui, 2022	NP	Surgical (scoliosis vs herniation)	Paraffin	IHC (brightfield)	MMPCC
Yang, 2018	IVD (inner AF, NP)	Cadaveric controls; degenerated (chronic LBP/dizziness)	Paraffin	Staining: H&E; IHC (brightfield)	S100, SP
Yang, 2019	IVD	n.r. (large cohort)	Paraffin	IHC (brightfield)	VDR
Chen, 2019	IVD	Surgical (trauma/deformation vs herniation)	Paraffin	IHC (brightfield)	PON1
Yang, 2020	IVD	Surgical (fractures vs herniation/spondylolisthesis)	Paraffin	Staining: H&E, Safranin O; IHC (brightfield)	S1PR1, S1PR2, S1PR3
Li, 2018	IVD	Surgical (scoliosis vs herniation; mild/severe)	Paraffin	IHC (brightfield)	WNT5a, TNFα
Lama, 2023	IVD	Cadavers / scoliosis / herniation (by grade)	OCT	Staining: H&E, Toluidine blue; IHC (brightfield)	MMP1, Caspase3, Ki67, PCNA
Binch, 2015	IVD	n.r.	Paraffin	IHC (brightfield)	Sema3C, Sema3D, NRP2, PA1
Nakazawa, 2018	IVD	Intact, non-herniated (graded)	MMA	IHC and IF	CCR7, CD163, CD206
Aras, 2016	IVD	Herniation (graded)	Paraffin	IHC (brightfield)	MMP11
Dube, 2025	IVD	n.r.	Paraffin	Staining: H&E; IHC (brightfield)	AEBP1
Wang, 2021	NP	Surgical (scoliosis vs herniation)	Paraffin	IHC (brightfield)	PIEZO1
Yu, 2022	IVD	Surgical (fractures/scoliosis vs n.r.)	Paraffin	IHC (brightfield)	ANG2
Zhu, 2025	IVD	Surgical (scoliosis/fractures vs herniation)	Paraffin	Staining: H&E, Safranin-O/Fast Green; IHC (brightfield)	POSTN, NLRP3, GSDMD-N, NOTCH1, IRF2
Liu, 2016	IVD	Surgical (scoliosis vs chronic LBP)	Paraffin	Staining: H&E; IHC (brightfield)	CD24, TIE2
Ionescu, 2024	IVD	Surgical (fractures vs herniation)	Paraffin	Staining: H&E; IHC (brightfield)	SIRT1

Guo, 2017	IVD	Surgical (trauma vs herniation)	Paraffin	IHC (brightfield)	MTH1
Zhang, 2022	IVD	Surgical (scoliosis vs herniation/spondylolisthesis)	Paraffin	Staining: H&E, Alcian blue; IHC (brightfield)	ENPP2, NOX4, FADS2
Zhang, 2023	IVD	Surgical (trauma vs herniation)	Paraffin	IHC (brightfield)	COLL II, ACAN, ADAMTS4, TNFα
Chen, 2022	IVD	Surgical (scoliosis vs n.r.)	Paraffin	IHC (brightfield)	COLL II
Lian, 2017	IVD	Surgical (scoliosis vs degenerative disc disease)	Paraffin	IF	ANGPTL8
Liao, 2019	NP	Surgical (scoliosis vs degenerative disc disease)	Paraffin	Staining: H&E; IHC (brightfield)	ADAMTS5, MMP3, MMP13, NLRP3, GSDMD, CASPASE1, IL1β, MINK1
Zhan, 2024	IVD	Surgical (LBP)	Paraffin	IHC (brightfield)	NRF2
Tang, 2019	IVD	Cadaveric / prolapse	Paraffin	Staining: H&E; IHC (brightfield)	AQPs 1, AQP 5
Johnson, 2015	IVD	Surgical (herniation)	Paraffin	IF	PIEZO1
Li, 2025	IVD	Surgical (fracture/herniation/stenosis)	Paraffin	IHC (brightfield)	ANG2, COLL II, MMP13
Wang, 2018	IVD	Surgical (mixed degenerative diagnoses)	Paraffin	IHC (brightfield)	LRP1
Yao, 2025	IVD	Surgical (chronic LBP)	Paraffin	IHC (brightfield)	SOX9
Kang, 2017	IVD	Surgical (herniation/stenosis)	OCT	Staining: H&E, Safranin O/Fast Green; IHC (brightfield)	p16, EZH2, pSTING, IL1β, IL6
Zheng, 2025	IVD	Surgical (fracture vs herniation)	Paraffin	IHC (brightfield)	SDF1, CXCR4
Jiang, 2019	CEP	Surgical (fracture vs degeneration)	Paraffin	IHC (brightfield)	EZH2
Ding, 2022	CEP	Surgical (scoliosis vs severe degeneration)	Paraffin	Staining: H&E, Alcian blue, Safranin O/Fast Green; IHC (brightfield)	YAP1, pYAP1, COLL II
Huang, 2020	CEP	Surgical (fracture vs chronic LBP)	Paraffin	Staining: H&E, Safranin O/Fast Green, Alcian blue; IHC (brightfield)	MMP13, COLL II, Substance P, TNFα
Huang, 2023	CEP	Surgical (fracture vs chronic LBP)	Paraffin	Staining: H&E, Alcian blue, Safranin O/Fast Green; IHC; IF	COLL II; NRF2
Chen, 2024	IVD	Surgical (LBP/hernia/stenosis/spondylolisthesis)	Paraffin	IHC (brightfield)	COX2, PGE2, EP4
Bing, 2024	IVD	Surgical (LVF vs IDD)	Paraffin	Staining: H&E, Safranin O/Fast Green; IHC (brightfield)	P16, P21, MMP13, COLL I, OCN
